# Crystal structures of ryanodine receptor SPRY1 and tandem-repeat domains reveal a critical FKBP12 binding determinant

**DOI:** 10.1038/ncomms8947

**Published:** 2015-08-06

**Authors:** Zhiguang Yuchi, Siobhan M. Wong King Yuen, Kelvin Lau, Ainsley Q. Underhill, Razvan L. Cornea, James D. Fessenden, Filip Van Petegem

**Affiliations:** 1 Department of Biochemistry and Molecular Biology, The Life Sciences Centre, University of British Columbia, 2350 Health Sciences Mall, Vancouver, Canada V6T 1Z3; 2 Department of Biochemistry, Molecular Biology and Biophysics, University of Minnesota, 321 Church Street SE, Minneapolis, Minnesota 55455, USA; 3 Department of Anesthesia, Perioperative and Pain Medicine, Brigham and Women's Hospital, 75 Francis Street, Boston, Massachusetts 02115, USA

## Abstract

Ryanodine receptors (RyRs) form calcium release channels located in the membranes of the sarcoplasmic and endoplasmic reticulum. RyRs play a major role in excitation-contraction coupling and other Ca^2+^-dependent signalling events, and consist of several globular domains that together form a large assembly. Here we describe the crystal structures of the SPRY1 and tandem-repeat domains at 1.2–1.5 Å resolution, which reveal several structural elements not detected in recent cryo-EM reconstructions of RyRs. The cryo-EM studies disagree on the position of SPRY domains, which had been proposed based on homology modelling. Computational docking of the crystal structures, combined with FRET studies, show that the SPRY1 domain is located next to FK506-binding protein (FKBP). Molecular dynamics flexible fitting and mutagenesis experiments suggest a hydrophobic cluster within SPRY1 that is crucial for FKBP binding. A RyR1 disease mutation, N760D, appears to directly impact FKBP binding through interfering with SPRY1 folding.

## Abstract (web summary)

The ryanodine receptor (RyR) is a large multi-domain ion channel that functions to release calcium from the endoplasmic or sarcoplasmic reticulum. Here the authors present crystal structures of the SPRY1 and tandem repeat domains of RyR, allowing precise positioning of the domains and linking disease mutations to RyR function.

## Graphical abstract

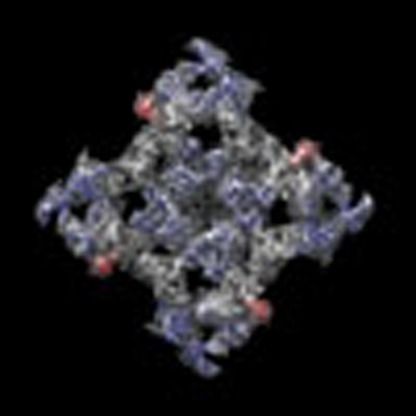

Ryanodine receptors (RyRs) form ∼2.2 MDa calcium release channels located in the membrane of the sarcoplasmic and endoplasmic reticulum. Triggered by either cytosolic or luminal Ca^2+^, or by conformational changes in the skeletal muscle dihydropyridine receptor, these proteins govern several Ca^2+^-dependent processes, including the contraction of skeletal and cardiac muscle^1^,^2^,^3^.

RyRs consist of multiple globular domains, interspersed by long α-helical regions (Fig. 1). Several studies by X-ray crystallography and NMR have revealed high-resolution structures of domains in the N-terminal region^4^,^5^,^6^,^7^,^8^,^9^,^10^, the SPRY2 domain^11^, and a phosphorylation hot spot within a tandem repeat domain^12^,^13^. As large proteins, RyRs represent ideal targets for cryo-EM studies^14^, which have shown that they form mushroom-shaped tetrameric assemblies. The stalk crosses the membrane, whereas the cap is located entirely in the cytosol. Three recent cryo-EM structures of RyR1 have been reported with resolutions up to 6.1 (ref. 15), 4.8 (ref. 16) and 3.8 Å (ref. 17). These studies have detected long α-helix-rich regions resembling armadillo repeats in the cytosolic cap, which form scaffolds for several other domains. The best electron density was obtained for the pore region, but due to the inherently dynamic structure of the cytosolic assembly, the local resolution in the cap falls down to 6.2 Å in the corners for the 3.8 Å map.

RyRs contain three different SPRY domains, named after ‘SPlA kinase and Ryanodine Receptor’ where they were first discovered. In other proteins, SPRY domains have been implicated in protein–protein interactions, but in RyRs their function is not well defined^18^,^19^. From the high-resolution cryo-EM structures, all three RyR SPRY domains appear to be located in the clamp region, but *de novo* tracing of the Cα backbone was not possible for these domains (Supplementary Fig. 1). Thus, all three groups placed homology-based models, which were then either left as is or adjusted manually. The exact positioning of each model was based on analysing the connectivity to other domains in the RyR, but due to inherent ambiguity at this resolution (Supplementary Fig. 1), different conclusions were reached on the positions of each domain.

RyRs bind many accessory proteins and small molecules that affect their gating properties allosterically. Among these are FKBP12 and FKBP12.6 (FK506 binding proteins of 12 and 12.6 kDa, respectively) that associate with RyRs with high affinity. FKBPs promote the closed state and decrease the appearance of subconductance states^20^. Several studies have implied that unbinding of FKBP12 or FKBP12.6 may contribute to diseases associated with RyR Ca^2+^ leak like muscular dystrophy, sarcopenia, heart failure, diabetes, or Alzheimer’s^21^,^22^,^23^,^24^. Although this phenomenon remains highly controversial, stabilizing FKBP-RyR interactions is a promising therapeutic route for these pathologies. For example, 1,4-benzothiazepine derivatives like JTV519 and S107 are thought to stabilize the FKBP–RyR interaction and have been found to improve function of heart and skeletal muscle in animal disease models (for example, refs 25, 26). As such, there is great interest in determining the structural elements responsible for FKBP12 and FKBP12.6 binding to RyRs, which would impact both the fundamental understanding of RyR structure–function correlations and RyR-targeted therapy development. Low-resolution cryo-EM studies^27^,^28^,^29^, as well as fluorescence resonance energy transfer (FRET) measurements^30^ show that FKBP12 and FKBP12.6 bind in the same position and orientation to the periphery of the cytosolic cap, at the junction between the ‘clamp’ and ‘handle’ domains. This binding mode has been confirmed with the higher resolution cryo-EM studies, which showed either FKBP12 (ref. 17) or FKBP12.6 (ref. 16) bound to the same locus on RyR1 (we will refer to both as FKBP throughout the manuscript). However, the exact binding determinants for FKBPs within the RyR protein sequence have remained ambiguous, as numerous sites have been identified throughout the channel^31^,^32^,^33^,^34^.

Here we show high-resolution structures of the SPRY1 domain, as well as a tandem-repeat domain (‘Repeat12’) located between the SPRY1 and SPRY2 domains (Fig. 1). Repeat12 displays a different fold from the phosphorylation domain (Repeat34), which changes the overall shape. Using the new cryo-EM maps, we confirm our previous location of the SPRY2 domain^11^, and show that the SPRY1 domain is located next to FKBP. We validate the SPRY1 position through FRET-based trilateration and site-directed mutagenesis, and show that a SPRY1 loop is a major FKBP binding determinant. A disease mutation in the SPRY1 domain affects FKBP binding through interfering with the folding.

## Results

### Structure of the RyR2 SPRY1 domain

We solved the crystal structure of the mouse RyR2 SPRY1 domain, encoded by residues 650-844 (equivalent to rabbit RyR1 residues 639–833) up to 1.2 Å resolution (Table 1, Fig. 2, Supplementary Fig. 2). The structure contains two chains in the asymmetric unit; because these are nearly identical, all analysis is performed on chain A. The structure reveals several features not observed in the recent cryo-EM studies, which have relied on homology-based models (Fig. 2b,c). These discrepancies cannot be ascribed to different isoforms, because the SPRY1 sequence is highly conserved among all three RyR isoforms (Fig. 2d). The core of the structure consists of two antiparallel β-sheets. A ‘lid’ region, also observed for the SPRY2 domain, forms a cap over this core. An insertion that creates a β-hairpin protruding from the core distinguishes SPRY1 from SPRY2. This ‘finger’ sticks out from the core, and is stabilized by the conserved Trp713 residue at the base that forms an anchor point. The conformation of this finger is identical in both molecules of the asymmetric unit and its residues are highly conserved among all three RyR isoforms (Fig. 2d), suggesting it plays a functional role by forming an anchor point for other RyR domains or auxiliary proteins. The SPRY2 domain contains an ‘insertion loop’, which breaks a β-strand typically observed in other SPRY domains in two halves^11^. This insertion loop is not observed in the SPRY1 domain. So, despite their very similar core fold, SPRY1 and SPRY2 have diverged via several insertions that affect their overall shape (Supplementary Figs 3a,4 and 5).

This crystal structure differs from the models proposed by the cryo-EM studies. Even for the highest resolution cryo-EM structure, there are many differences (for example, see the RMSD plot in Fig. 2c). The β-hairpin forming the ‘finger’ is lacking and has instead been modelled as a loop, devoid of secondary structure, pointing in a different direction (root mean-squared deviation (r.m.s.d.) values >20 Å, Fig. 2b,c). In addition, there is a two-step shift in the sequence register for two loops and a core β-strand, resulting in r.m.s.d. values of ∼7 Å. Such shifts in register cannot be explained by sequence differences between RyR1 and RyR2, as the SPRY1 domain is highly conserved (Fig. 2d). Although these new cryo-EM studies are a substantial advance in the field, it is premature to assume positional correctness of every amino acid in the model, even those originating from the highest resolution map.

### Structure of the tandem repeats

**Figure 1:**
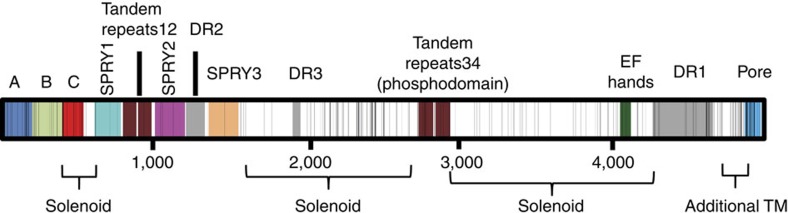
Linear overview of the RyR1 sequence. Vertical lines represent disease-associated mutations. RyR1 consists of a large scaffold of several α-solenoid regions, interspersed by globular domains (coloured) and three ‘divergent regions’ (DRs) of high sequence dissimilarity among isoforms. Domain C (red) is part of a larger solenoid. The current study documents the atomic structure and docking of the SPRY1, tandem Repeat12 and SPRY2 domains.

The SPRY1 and SPRY2 domains are separated in sequence by tandem repeats, encoded by RyR1 residues 862-1054 (‘Repeats12’; Fig. 1). We solved the crystal structure of RyR1 Repeat12 at 1.5 Å resolution (Fig. 3), which shows that its two halves are not symmetrical. Each repeat consists of two α helices, and short single β-strands at each C terminus assemble to form a two-stranded β-sheet. However, Repeat2 also contains an extra three-stranded β-sheet which breaks the symmetry. This sheet is formed at the expense of its first α helix (α_1_’), which is considerably shorter than the corresponding helix in Repeat1 (α_1_). The β-sheet fills up the space between Repeat1 and Repeat2 and mediates extra interactions, including a salt bridge between Arg1,000 and Glu917 (Fig. 3a). The two Repeats are separated by an unusual 30-residue loop that is completely structured. This loop forms a large U-shaped lid (‘U-lid’) that makes intimate interactions with the α-helices of Repeat1.

This structure differs from previous crystal structures of the phosphorylation domain (also known as ‘Repeats34’ encoded by RyR1 residues 2734-2940)^12^,^13^ (Supplementary Figs 3b,4 and 6). Repeat34 is centrally located in the RyR sequence and forms a phosphorylation hot-spot domain that displays high pseudosymmetry and a prominent horseshoe shape. The loops linking the α-helices have different conformations, and the angle between the two repeats is wider for Repeat34. Importantly, it lacks the three-stranded β-sheet and the structured U-lid. Instead, Repeats 3 and 4 are separated by an unstructured linker that contains multiple phosphorylation sites in RyR1 and RyR2 (ref. 12). These differences between Repeat12 and Repeat34 change the overall surface of these two domains, whereby Repeat12 no longer displays a horseshoe shape (Supplementary Fig. 4).

The U-lid and the three-stranded β-sheet for the Repeat12 domain were not resolved in any of the recent cryo-EM studies of intact RyR1 (refs 15, 16, 17). The local resolution is different throughout various regions, and was found to be lowest in the corner region, estimated at ∼6.2 Å for the 3.8 Å map. The Repeat12 domain, which is thought to reside in this corner, was therefore not traced *de novo*, but modelled based on the Repeat34 structures. As a result, these models differ substantially from the high-resolution crystal structure we report here, with r.m.s.d. values of ∼10 Å for the modelled portion of the U-lid, and up to 20 Å in the β-sheet region (Fig. 3b,c).

### Docking in RyR1 cryo-EM maps

Since the recent high-resolution cryo-EM studies obtained conflicting results concerning the locations of the SPRY domains, we decided to dock these two new crystal structures, as well as our previously reported SPRY2 structure^11^ into the three new cryo-EM maps of RyR1 (refs 15, 16, 17). We used unbiased six-dimensional searches as implemented in ADP_EM^35^, which yielded identical results (location and relative orientation) in each of the three recent cryo-EM maps (Fig. 4, Supplementary Figs 7 and 8). The position and orientation for the SPRY2 domain are identical to a previous docking we reported using lower resolution cryo-EM maps^11^. The SPRY1 domain is located right next to FKBP, and the top hit for Repeat12 is in the corner.

We and others have previously docked the Repeat34 domain to the corner^12^,^13^, now occupied by the Repeat12 domain. Despite the improved resolution of the maps, direct docking of the Repeat34 domain still yields the corner position as the top hit, with very similar correlation coefficients (Supplementary Fig. 9). However, direct placement of Repeat12 or Repeat34 in the turret location, followed by rigid-body refinement in Situs^36^, yields a significantly higher correlation coefficient for Repeat34 (Supplementary Fig. 9). Together with the assignment of flanking sequences to an area contacting the turret, the Repeat34 domain is most likely located within the turret, where the density is very poor and unbiased docking subsequently fails. This automatically only leaves the corner position for Repeat12, although there remains a visible mismatch because the cryo-EM density does not support the three-stranded β-sheet (Fig. 4c, Supplementary Fig. 9). This mismatch, likely due to the intrinsic mobility of the corner region, suggests that further experimental proof or higher resolution cryo-EM data may be required to unambiguously resolve the Repeat12 and Repeat34 positions.

Visual inspection is sufficient to determine that the crystal structure of SPRY1 fits much better than SPRY2 next to FKBP, a feature supported by plots of the correlation coefficients per secondary structure element (Supplementary Fig. 10).

### FRET-based localization of the RyR1 SPRY1 domain

To validate our docking of SPRY1, we conducted FRET-based trilaterations using a set of FKBPs labelled with AlexaFluor488 (AF488, donor), which can undergo distance-dependent energy transfer to a Cy3NTA (acceptor), directed to deca-histidine (His_10_) ‘tags’ inserted in the SPRY1 domain. Informed by our RyR2 SPRY1 atomic structure, we inserted His_10_-tags into two separate surface-exposed loops at equivalent positions 655 and 675 in rabbit RyR1 (RyR constructs His^655^ and His^675^). Full-length RyR1 constructs containing each His_10_-tag insertion expressed in HEK-293T cells exhibited no significant change in caffeine-induced calcium release relative to wild-type (WT)-RyR1 (Supplementary Fig. 11).

We targeted each of the five AF488-FKBP donors to these His10-tagged RyRs (expressed in HEK cells) and measured FRET to Cy3NTA acceptor attached at the His_10_ tag. FKBP binding to His^675^ was reduced to less than 10% relative to WT RyR1, suggesting that this site plays an important role in FKBP/RyR binding. Consequently, FRET could not be measured for this construct. However, robust FRET from all five FKBP donor positions (highest from D32, and lowest from D49) was observed to Cy3NTA targeted to His^655^ (Fig. 5), thus allowing us to readily determine a discrete locus for Cy3NTA bound to His^655^. This locus overlaps the 655 loop within the docked SPRY1 domain but is >40 Å from the 655 loop predicted from an alternatively proposed position for SPRY1 (refs 15, 16; Fig. 5; Supplementary Fig. 12). This result independently validates our docking solution for SPRY1 in direct contact with FKBP.

Two of the recent cryo-EM studies^15^,^16^ assigned SPRY2 to the density next to FKBP, whereas one assigned this to SPRY1 (ref. 17). Our docking and trilateration thus confirm the latter study, but a detailed analysis of the SPRY1–FKBP interface from this cryo-EM study alone is not possible, due to errors in the sequence register which affect one of the loops at the interface. On the other hand, it is very likely that some loops at domain–domain interfaces adopt different conformations from the ones seen in our crystal structure. We therefore decided to use our docked SPRY1 crystal structure as a starting point for molecular dynamics flexible fit (MDFF) experiments.

### Molecular dynamics flexible fit

MDFF constrains MD calculations within the context of a cryo-EM map. The neighbouring SPRY2 and FKBP were included to constrain the conformational freedom of SPRY1 and to allow optimization of the SPRY1–FKBP interface. The overall map-correlation coefficient improved relative to the previously proposed model^17^ (Fig. 6). Importantly, loops at the FKBP interface show an improved match between model and map.

We analysed these MDFF results in the 3.8 and 4.8 Å maps in the context of the SPRY1–FKBP interface. In both cases, the results implicate an interface burying ∼350 Å^2^, primarily due to 6–7 SPRY1 residues in three different loops (Fig. 6, Supplementary Table 1). All three loops show improved local fits in the map (Fig. 6c). One loop, containing L719, is located within the region containing the double shift in sequence register and so was previously not found in VDW contact in the 3.8 Å cryo-EM model (Supplementary Table 1). A second loop implicates residues H736 and L737, but the largest contact with FKBP seems to occur with the 675 loop, with the F674 side-chain fitting into a pocket formed by two arginines on the FKBP surface. One of these, R40, is now positioned perfectly for a cation-pi interaction with the F674 side chain. The fit in the 4.8 Å map also suggests a possible involvement of L675, in direct interaction with both F674 and FKBP. Although the precise interactions remain to be observed via a higher resolution study of the SPRY1–FKBP interface, we decided to experimentally validate the result using FKBP–RyR1 binding assays.

### FKBP–RyR1 binding assays

To quantify the effects of inserting His_10_-tags in the SPRY1 domain (of the full-length RyR1) on FKBP binding, we created N-terminal GFP fusions of each of these RyR1 constructs and then determined FKBP binding affinity (*K*_d_) and off-rate kinetics (*k*_off_) using an AlexaFluor568-labelled FKBP (AF568–FKBP). AF568–FKBP bound similarly to GFP–WTRyR1 and GFP–His^655^, whereas markedly diminished binding was observed to GFP–His^675^ (Supplementary Fig. 14). The His^675^ insertion dramatically increased the FKBP–RyR1 *K*_d_ (reduced the binding affinity), whereas for FKBP binding to GFP–His^655^ neither *K*_d_ nor *B*_max_ were significantly changed relative to GFP-RyR1 (Fig. 7a). Changes in FKBP binding affinity for GFP–His^675^ were most likely driven by the faster *k*_off_ observed in dissociation experiments (Fig. 7b). A partial scramble of only five amino-acid residues in the RyR1 SPRY1 675 loop had similar effects on *K*_d_ (Fig. 7c) and *k*_off_ (Fig. 7d) as the His_10_ insertion at position 675. Since the MDFF experiments suggest a crucial involvement of either a single Phe or Phe–Leu cluster within the 675 loop, we mutated both residues to Ala in the full-length RyR1. This double Ala substitution completely replicated the effects on *K*_d_ and *k*_off_ of either His^675^ insertion or scrambling of the SPRY1 675 loop (Fig. 7c,d), thus suggesting that these hydrophobic residues play a crucial role in FKBP–RyR1 binding.

These findings are in excellent agreement with the proposed SPRY1 domain location. The loop containing residue 675 is located directly at the interface with FKBP and thus explains the drastic effect of either a His_10_ insertion or loop scrambling on FKBP binding. The His_10_ insertion at site 655 is spatially located near the interface between SPRY1 and Repeat12 (Figs 4, 5c), but appears accessible in the full-length RyR1, explaining why the insertion has no effect on FKBP affinity. Thus, these results are consistent with our proposed new location for the SPRY1 domain, and suggest that the SPRY1 domain is a crucial interaction site for FKBP.

Several RyR sequence elements have previously been suggested as FKBP binding determinants, including a putative PKA phosphorylation site (S2843) and a conserved VP dipeptide at position 2461 (refs 21, 31). We re-evaluated FKBP binding to RyR1 containing mutations in these previously proposed sites. The S2843D phosphomimetic or the S2843A phosphorylation-null mutations had no significant effect on FKBP binding in our assay (Supplementary Fig. 15). A second mutation, V2461G, had no effect on *K*_d_ but a significant effect on *B*_max_ (Supplementary Fig. 15). However, according to the new cryo-EM maps^15^,^16^,^17^, both S2843 and V2461 are located far (>60 Å) from FKBP (Supplementary Fig. 15b), suggesting that FKBP binding is allosterically affected by the point-mutation at position 2,461. This allosteric effect is minor compared with mutations in the SPRY1 675-loop.

### Disease-causing mutations

The SPRY1 and Repeat12 crystal structures allow us to look at the detailed environments for amino acids linked to genetic disorders (Figs 8 and 9). The Repeat12 domain boasts positions for five malignant hyperthermia mutations in RyR1 and two CPVT mutations in RyR2. Several of these are at the surface (Fig. 3a), so are unlikely to cause misfolding. However, the RyR1 R1043C mutation^37^ (R1044C in rabbit RyR1) affects a residue that is involved in multiple hydrogen bonds, including main chain atoms from the U-lid (Fig. 8b). Breaking the interaction may affect the U-lid conformation and stability. To investigate this further, we prepared purified mutant Repeat12 domain and compared the thermal stability with the WT domain (Fig. 8c). Whereas the WT shows a single transition at 41.1 °C, the R1044C mutant shows two transitions with *T*_m_ values at 33.6 and 39.6 °C, suggesting destabilization of a subdomain. The G1049S mutation^38^ (G1050S in rabbit RyR1) is located in a tight loop near the end of Repeat12. The main chain conformation is in a region of the Ramachandran plot that is only allowed for glycine residues. Thermal melt analysis does not suggest destabilization (*T*_m_ 43.1 °C), but the yield of purified recombinant protein is drastically lower (∼40-fold compared to WT), suggesting that a significant portion is misfolded during recombinant expression. RyRs have been shown to form 2D checkerboard lattices in their native environment^39^,^40^, and 2D crystallization experiments suggest that the corner region is involved in the crystal contacts^41^. Mutations on the surface of the Repeat12 may therefore interfere with inter-RyR contacts.

**Figure 2:**
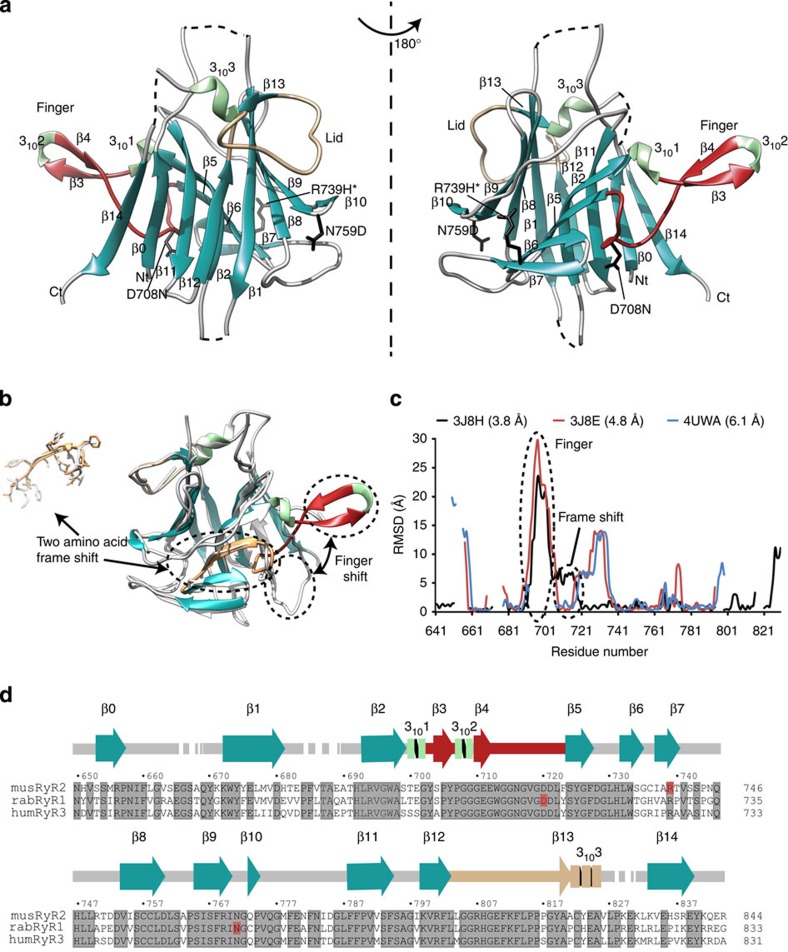
Crystal structure of the SPRY1 domain. (**a**) Two different views of the mouse RyR2 SPRY1 domain. β-strands are indicated in cyan and 3_10_ helices in light green. The ‘finger’, formed by a β-hairpin pointing away from the core is indicated in red. The lid following the core is indicated in beige. Positions for disease-associated mutations are shown in black sticks and labelled. Unstructured loops are indicated by dotted lines. (**b**) Superposition of the SPRY1 crystal structure (colours) and one of the models (white) proposed from cryo-EM studies (3.8 Å map). The main differences are highlighted. The 15-residue stretch containing a two-amino-acid shift in the register is highlighted in orange. (**c**) Plot showing the root mean-squared deviation (r.m.s.d.) per residue for the proposed SPRY1 models from three independent cryo-EM structures relative to the crystal structure. (**d**) Sequence alignment for the SPRY1 domain from RyR1, RyR2 and RyR3. Secondary structure elements for the RyR2 SPRY1 structure are indicated above the sequence. Disease-associated mutations are highlighted in red in the sequence. The ‘finger’ substructure is highly conserved among all three isoforms.

**Figure 3:**
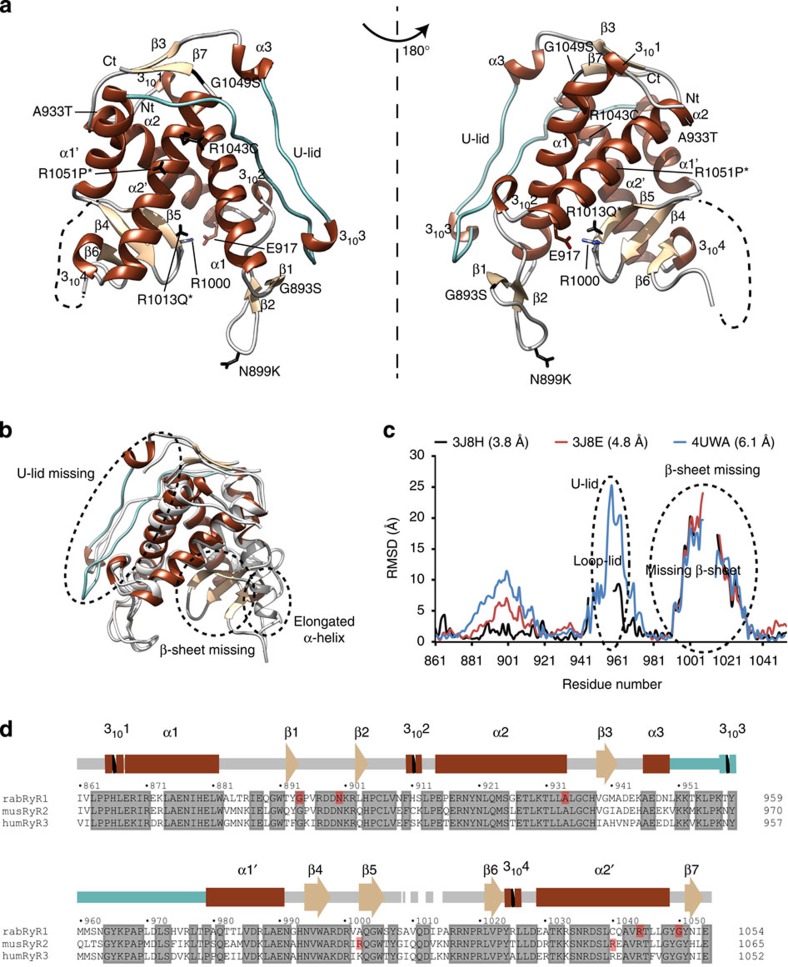
Crystal structure of the Repeat12 domain. (**a**) Cartoon representation of the rabbit RyR1 Repeat12 domain, showing α helices in brown and β- strands in beige. A long U-shaped and structured loop (‘U-lid’) that connects the two repeats is indicated in cyan. Positions for disease-associated mutations are shown in black sticks and labelled. A three-stranded β-sheet fills up the space between the two halves and interacts with the first half, in part through a salt bridge between Glu917 and Arg1000. (**b**) Superposition of the Repeat12 Domain crystal structure with a model proposed from cryo-EM studies (3.8-Å map). The latter model misses the U-lid and the three-stranded β-sheet. In addition, an alpha helix in Repeat2 is too long by 14 residues. (**c**) Plot comparing the r.m.s.d. values per residue for all previously proposed Repeat12 models relative to the crystal structure. The largest differences are highlighted. (**d**) Sequence alignment for the Repeat12 domain from RyR1, RyR2 and RyR3. Secondary structure elements for the RyR1 Repeat12 structure are indicated above. Disease-associated mutations are highlighted in red.

Within SPRY1, three mutations have previously been linked to disease (Fig. 2a). The human RyR1 mutation D708N has been linked to multi-minicore disease and atypical periodic paralysis^42^. The corresponding residue in RyR2, Asp720, forms a salt bridge with R694 (Fig. 8a), which is also conserved among the RyR isoforms. Asp720 is located on the finger, which protrudes from the SPRY1 core and which mediates interactions with the SPRY2 domain. Melting analysis shows only a slight destabilization of the thermal stability. Although the effect is small (∼1 °C lower), the WT domain already has a low *T*_m_ in isolation (∼40 °C, corresponding to ∼30% unfolding at 37 °C), so small changes could impact stability at physiological temperatures. We postulate that the primary effect of the mutation is on the SPRY1–SPRY2 interaction.

### N760D affects FKBP binding

Given the role for the SPRY1 domain in FKBP binding, one could expect that some mutations act primarily by affecting the FKBP affinity. No disease mutations have yet been identified directly at the SPRY1:FKBP interface, but the central core disease mutation N760D (N759D in human RyR1)^43^ may indirectly affect FKBP binding. The corresponding residue in RyR2 (Asn771) is involved in a hydrogen bond network that involves Asp753 and His747 (Fig. 9a), the latter of which is directly in contact with FKBP (Fig. 6, Supplementary Table 1). These residues are conserved among all three RyR isoforms, suggesting the network also exists in RyR1. The N760D mutation is likely to cause repulsion between the two negatively charged Asp residues, thus leading to conformational changes in this area close to FKBP.

To further investigate this, we performed FKBP binding studies and found N760D to cause a 74±5% reduction in *B*_max_ but with no significant effect on the affinity (Fig. 9b) or *k*_off_ (Fig. 9c). Given this surprising result, we took a closer look at this mutation within the isolated RyR1 SPRY1 domain. Multiple trials of expression and purification of this mutant consistently led to yields that were four- to eightfold lower compared with WT (Fig. 9d), suggesting a significant impact on folding. The purified protein has a melting temperature that is ∼3.5 °C lower than WT (Fig. 8c; 36.6 versus 40.1 °C), which does not explain the lower yield since the recombinant expression and purification were performed at 18 °C and 4 °C, respectively. Size exclusion chromatography does not show signs of aggregation, suggesting the purified protein is folded. These observations are consistent with the effects on FKBP binding, with lowered *B*_max_ due to ∼75% misfolded SPRY1, but unaltered affinity for the remaining ∼25% that does fold properly. We hypothesize that the N760 residue thus plays a role within the folding pathway of the SPRY1 domain. Importantly, these data show for the first time that a disease mutant can have a profound effect on FKBP binding.

## Discussion

RyRs have been the topic of intense structural investigation using both high- and low-resolution methods^3^,^14^, and recent cryo-EM studies have reported the RyR1 structure with resolutions of 6.1-3.8 Å (refs 15-17, 16, 17). However, at these resolutions, tracing the amino acid backbone *de novo* can still be challenging, particularly for regions devoid of α helices and in regions with lower local resolution. RyRs contain five domains that consist predominantly of β-sheets: two β-trefoil domains encoded by the N-terminal ∼400 residues (also named domains A and B), and three SPRY domains located within the 633–1600 region (SPRY1-3). As such, these domains still provide a challenge to be identified through *de novo* tracing.

A visual inspection of the new RyR1 maps shows that there are only five distinct globular regions per subunit that are devoid of α helices, which should naturally correspond to these five domains. Two of these are located proximal to the fourfold symmetry axis, and were previously assigned to the β-trefoil folds of domains A and B^5^,^7^. The corresponding N-terminal domains in the IP_3_ receptor were found to occupy the similar region next to the fourfold symmetry axis, highlighting the similar architecture and evolutionary relationships between both classes of Ca^2+^ release channels^2^,^3^,^44^. These positions have been validated by difference cryo-EM and FRET studies^33^,^45^, and have been confirmed in the new maps^15^,^16^,^17^. Consequently, the three remaining regions, which are found within the clamp region, should correspond to the three SPRY domains. Correspondingly, IP_3_ Receptors, which do not have clamp regions^46^, also do not have SPRY domains in their sequence. Since crystal structures were not available at the time, the authors of all three recent cryo-EM reports generated homology-based models and placed them based on apparent connectivity to the armadillo repeat region following domain C. However, different pathways of connectivity are apparent in each map, resulting in ambiguity for the location of each SPRY domain (Supplementary Fig. 1).

**Figure 4:**
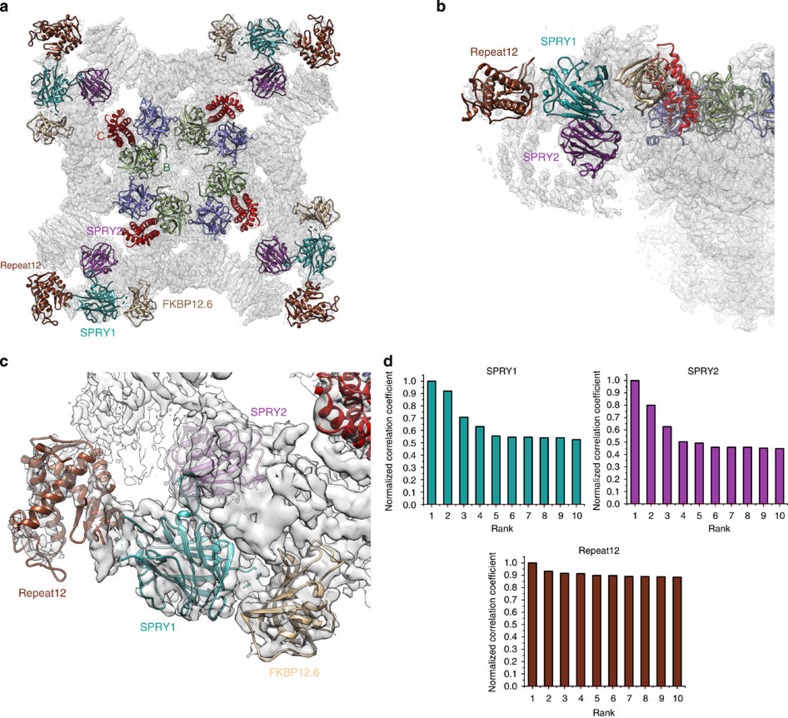
Positions of SPRY1, Repeat12 and SPRY2 in full-length RyRs. (**a**) Overall ‘top’ view facing the SR membrane from the cytosol, (**b**) Close-up side view, and (**c**) Close-up top view of several RyR domain crystal structures in the 3.8-Å cryo-EM map of rabbit RyR1 (EMDB 2807). Different domains are indicated in different colours. The positions for the N-terminal domains (A,B,C) are also shown for reference. (**d**) Normalized correlation coefficients of the top 10 hits for docking of the SPRY1, Repeat12 and SPRY2 domains in the 3.8-Å map. Docking in two other recent maps at 4.8 and 6.1 Å yields identical results (Supplementary Figs 7 and 8).

**Figure 5:**
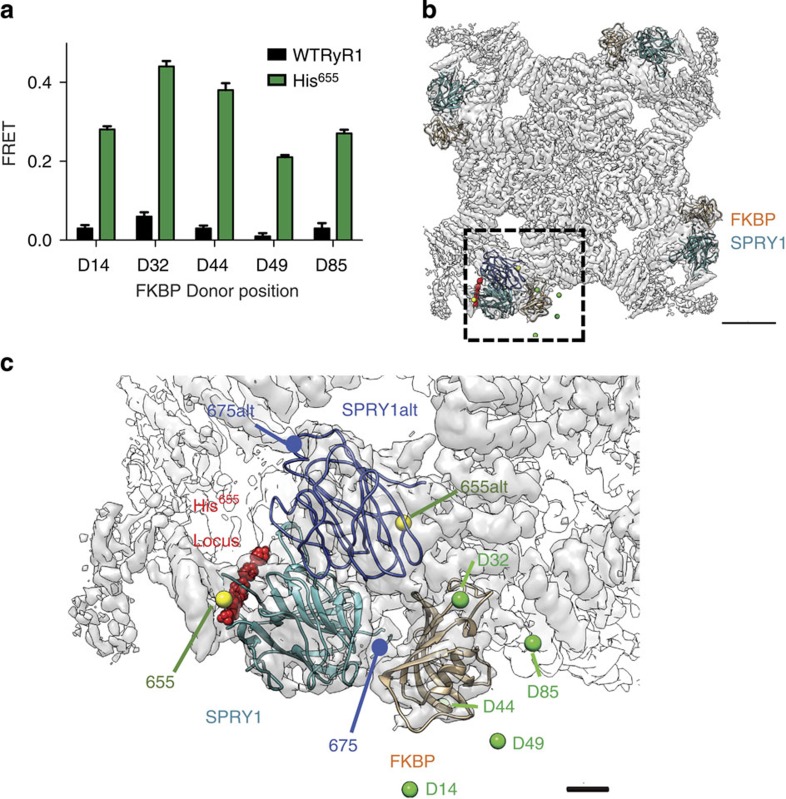
Trilateration of the RyR1 SPRY1 domain. (**a**) FRET efficiencies from each of the five AF488 donors covalently bound at the specified FKBP positions, to 3 μM Cy3NTA targeted to HEK-293T cells expressing WT RyR1 (black bars), or RyR1 containing a His_10_-tag insertion at position 655 in SPRY1 (His^655^, green). Values represent mean±s.e.m. for *n*=27–36 cells as indicated in Supplementary Fig. 13. (**b**,**c**) His^655^ trilateration locus (red spheres) shown relative to atomic structures docked to the RyR1 cryo-EM map (EMDB 2807) viewed from the top (that is, from the t-tubule membrane *in vivo*). Trilateration locus is only depicted relative to one of the four RyR1 subunits for clarity. Insertion sites of the His_10_-tags within the docked SPRY1 domain as well as the locations of the five AF488–FKBP FRET donors used for trilaterations (green spheres) are shown in **c**. The positions for the 655 loop and 675 loop in SPRY1 are indicated. Also shown is the SPRY1 location from two recent cryo-EM studies (blue chain, SPRY1alt), and the corresponding position of the 655 and 675 loops (model taken from Zalk *et al*.^16^). Scale bars, 50 Å (panel **b**) and 10 Å (panel **c**).

**Figure 6:**
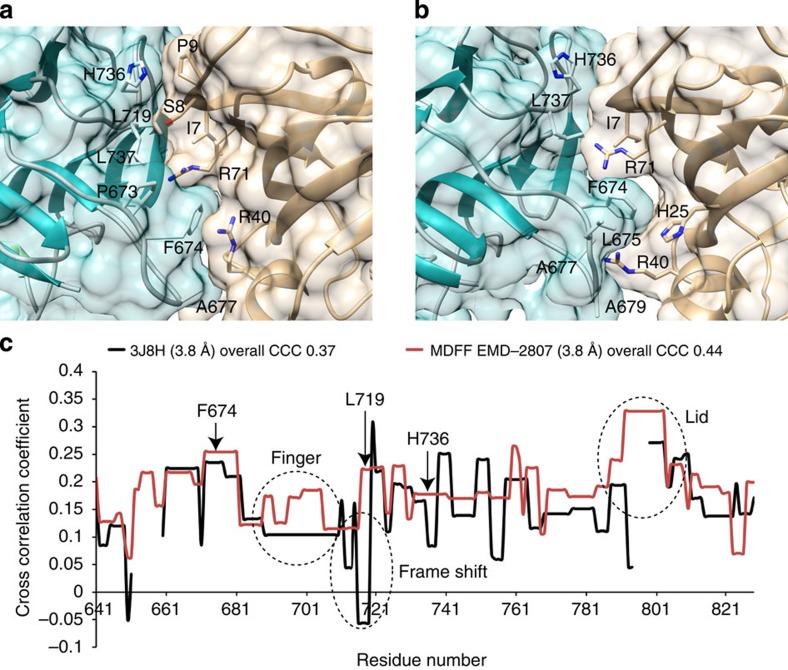
Molecular dynamics flexible fit of the SPRY1 domain. (**a**) Detail around the SPRY1–FKBP12 interface as obtained by MDFF in the EMD-2807 map (3.8 Å). The SPRY1 domain is shown in cyan and FKBP12 in beige. Labelled residues correspond to those that are in VDW contact at the interface as analysed by the software Chimera^52^. The interface buries 336 Å^2^. (**b**) Similar as in A, for the MDFF result in the EMD-6107 map (4.8 Å). The interface buries 391 Å^2^. F674 is involved in both cases. (**c**) Cross-correlation coefficients per secondary structure element, calculated by VMD^56^, for the proposed SPRY1 model from the 3.8 Å cryo-EM study (black) and the MDFF result obtained in the same map (red). The results vary per region, but the MDFF improved the overall correlation coefficient. Select residues at the interface are indicated for reference, as well as areas with major improvements.

**Figure 7:**
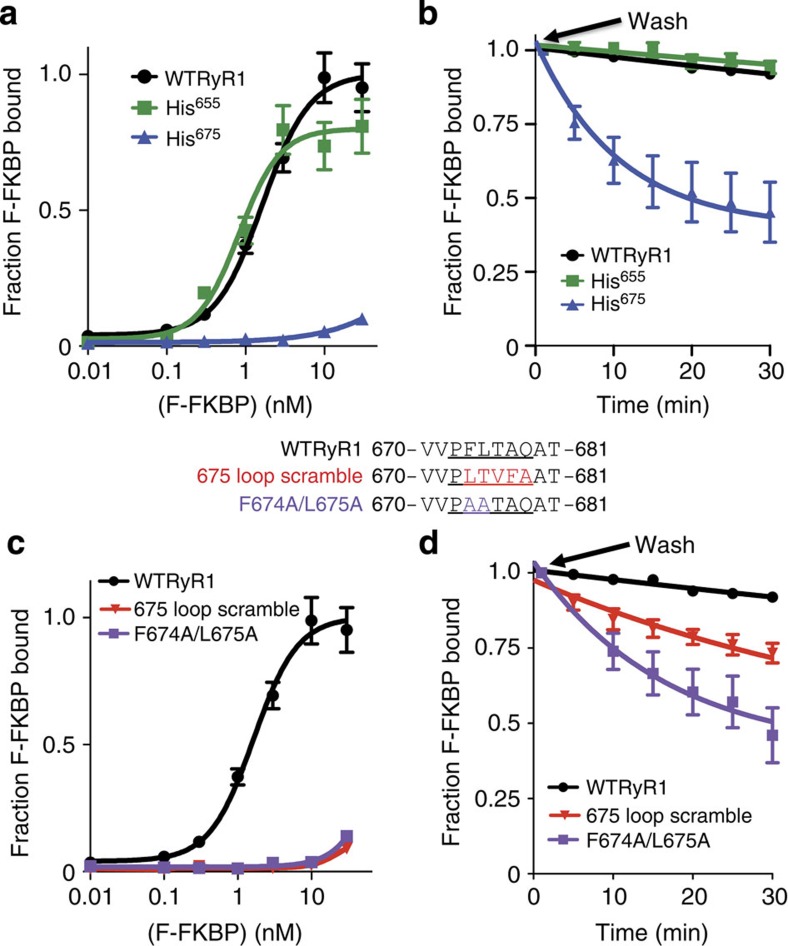
Analysis of FKBP binding to full-length RyR1 SPRY1 domain constructs. (**a**) Concentration dependence of AF568–FKBP (denoted F-FKBP) binding to the indicated GFP-fused RyR1 mutants expressed in HEK-293T cells relative to GFP-fused WT RyR1 (black symbols). (**b**) Time-course of AF568 F-FKBP dissociation after washing from HEK-293T cells expressing the indicated GFP-RyR1 fusion constructs. Curves represent single-exponential decay fits to the data. (**c**,**d**) Concentration dependence of AF568 F-FKBP binding and dissociation curves for GFP-fused RyR1 WT and mutant constructs containing the indicated scrambled sequence or double alanine substitution within the SPRY1 675 loop. For all panels, values represent mean±s.e.m. for *n*=16–41 cells as indicated in Supplementary Fig. 14.

**Figure 8:**
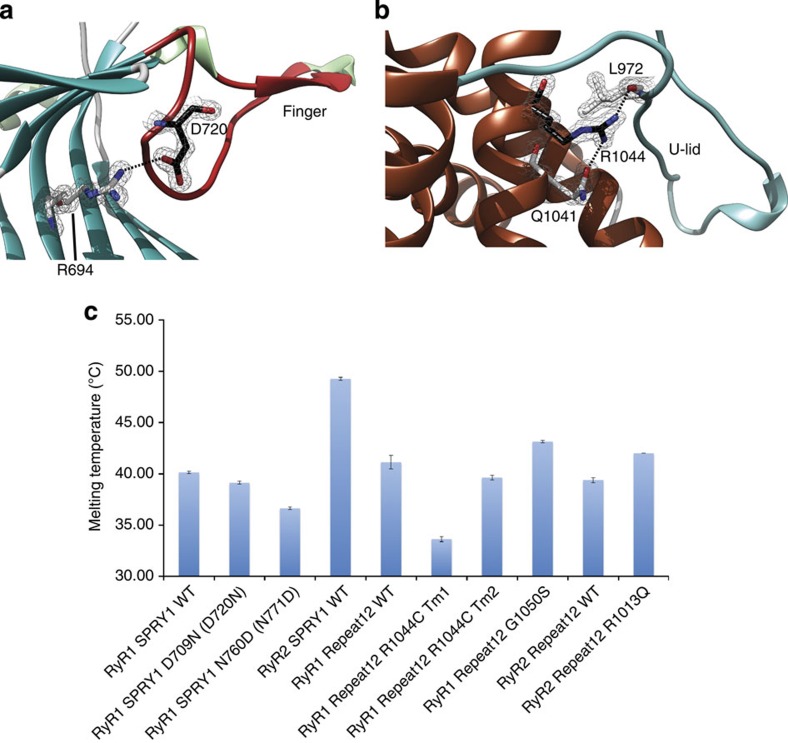
Disease-associated mutations in the SPRY1 and Repeat12 domains. (**a**) Detail around RyR2 D720, corresponding to the RyR1 D708N mutation linked to multi-minicore disease. D720 is located within the ‘finger’ (red) and makes a salt bridge interaction with R694. Both residues are conserved in RyR1-3. (**b**) Detail around rabbit RyR1 R1044, which corresponds to the human R1043C malignant hyperthermia mutation. R1044 is part of a hydrogen bond network that includes the main chain of the U-lid. (**c**) Melting temperatures for WT SPRY1 and Repeat12 domains, together with select disease mutants. Error bars correspond to standard errors, with *n*=4 for each. Note: the R1044C melting curve shows two transitions, resulting in two different melting temperatures.

**Figure 9:**
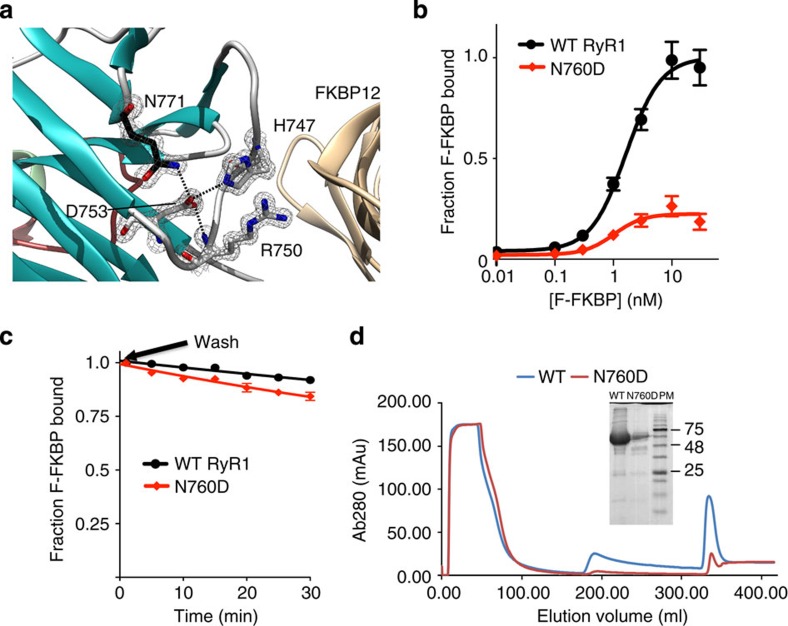
The N760D disease-linked mutation affects folding and FKBP binding. (**a**) Details around RyR2 SPRY1 N771, which corresponds to rabbit RyR1 N760D or human RyR1 N759D linked to central core disease. Electron density is shown around residues in the vicinity of N771, which is involved in a network of hydrogen bonds (dotted lines). The relative position of FKBP12 (beige) according to the SPRY1 docking is shown for reference. (**b**) Concentration dependence of AF568–FKBP (F-FKBP) binding to the GFP-fused N760D RyR1 construct (corresponding to the human N759D mutant; red symbols) relative to WT RyR1 (black symbols). (**c**) Time course of AF568 F-FKBP dissociation after washing from HEK-293T cells expressing the indicated GFP-RyR1 fusion constructs. Curves represent single-exponential decay fits to the data. Values represent mean±s.e.m. for *n*=16–60 cells as indicated in Supplementary Fig. 14. (**d**) Chromatogram of the rabbit RyR1 SPRY1 WT and N760D mutant, showing the results of the first Ni^2+^ affinity column (as HMT fusion, see methods). The last peak corresponds to the elution, which is eightfold lower in this example. The inset shows the corresponding SDS–PAGE of the elution fraction.

Having crystal structures of several new domains at hand, we utilized unbiased six-dimensional docking of the SPRY1, Repeat12 and SPRY2 domains. We find that SPRY1 is located next to FKBP, compatible with previous insertion studies of GFP near the SPRY1 sequence^47^, and SPRY2 one layer below the top surface, where we had localized it previously^11^ (Fig. 4). In addition, our FRET-based trilateration of SPRY1 in intact RyR1 supports our docking results, but is incompatible with an alternatively proposed location from two of the recent cryo-EM reports^15^,^16^. Interestingly, a loop that was found to be unstructured within the individual SPRY1 domain crystal structure appears structured in the maps containing FKBP (Supplementary Fig. 10), suggesting that FKBP binding induces structure in this disordered region. We performed MDFF experiments in the 3.8 and 4.8 Å maps, which point to either a Phe or Phe/Leu cluster in this loop that appears to make intimate interactions with FKBP, likely involving a cation-pi interaction between F674 in SPRY1 (rabbit RyR1) and R40 in FKBP12. In agreement with this, either scrambling of this loop, substitution of several residues by His_10_, or simple mutation of the Phe–Leu sequence virtually abolishes FKBP binding. The combined results of unbiased docking, trilateration, and FKBP binding make the SPRY1 position unambiguous, and show that the SPRY1 domain is a crucial contributor to FKBP binding.

The Repeat12 domain separates the SPRY1 and SPRY2 domains in the RyR sequence. Contrary to expectations, this domain has a shape that is very different from the phosphorylation domain (also known as Repeat34) that is located within the central region of the RyR sequence. This is largely due to the presence of an additional three-stranded β-sheet that fills up the space between the two halves of Repeat12. As a result, the phosphorylation domain has a prominent horseshoe shape, whereas Repeat12 does not (Supplementary Fig. 4). Previous docking of the phosphorylation domain in lower resolution maps put it at the location of the very corner, which also has a horseshoe-shaped density^12^.

Using unbiased docking in the new maps, the Repeat12 crystal structure docks to the corner region, despite the absence of the prominent horseshoe shape. Interestingly, unbiased docking of the phosphorylation domain in the new cryo-EM maps still yields the corner position as the highest hit. However, based on the assignment of alpha solenoids in the RyR1 central region, the phosphorylation domain is likely located within a flexible turret with poor density. In agreement with this, the correlation coefficient for Repeat34 in the turret is higher than for Repeat12 in this location. The discrepancy for the poor match in shape of Repeat12 at the corner is likely the result of the local resolution: although the highest resolution map has been estimated at 3.8 Å, the local resolution in the corner region was found to be closer to 6.2 Å^17^. A possible reason for the decreased resolution is that this area is very dynamic: indeed, it has been found that this area undergoes large movements associated with channel opening^15^,^29^, and this may have yielded incomplete density in this area. However, an unambiguous assignment of the Repeat12 and Repeat34 domains may benefit from higher resolution studies or experimental validation.

The advent of higher resolution cryo-EM studies on RyR1 clearly allows the unambiguous location of individual domains solved by X-ray crystallography. However, relying solely on the EM data to locate individual amino acids is still premature, even for the highest resolution cryo-EM studies currently available. In addition to the missing features for the Repeat12 domain, the proposed models for the SPRY1 and SPRY2 domains also differ substantially from the respective crystal structures. Although one could expect some differences in the conformation of loops at domain–domain interfaces, many of the observed discrepancies cannot be reconciled. Even for the 3.8 Å cryo-EM structure, the SPRY1 domain model has two-position shifts in the sequence register for two loops and a core β-strand, and the SPRY2 domain has a one-position shift in the register for the lid region. These shifts in register alter the chemical environment of the amino acids, and would lead to completely different interpretations of mutagenesis data. Side chains are completely invisible at the local EM resolution around the SPRY domains (for example, Supplementary Fig. 1, Fig. 4c), so more high-resolution studies are required to obtain a full atomic-scale picture of the RyR.

Our FKBP binding and docking studies implicate a hydrophobic cluster within a SPRY1 loop as a major FKBP binding determinant. Although it is clear from the cryo-EM maps that several other parts of the RyR1 protein should contribute to FKBP binding, these do not appear sufficient to overcome the loss of the interaction at RyR1 loop 675. Although previous studies have suggested involvement of very different sites, one must take into account that FKBP binding to RyR1 is most likely quite sensitive to allosteric effects since the new high-resolution cryo-EM maps show that a previously established FKBP motif at position 2,461 (ref. 31) is over 65 Å from the bound FKBP. The sensitivity of FKBP binding to allosteric changes most likely has led to confounding results from different groups as to the exact location of the FKBP binding site within the RyR primary sequence. The availability of high-resolution crystal structures of FKBP12/12.6 and SPRY1, together with the current constraints of cryo-EM maps, may facilitate the development of small molecules binding near the SPRY1–FKBP interface that can strengthen their interactions and have therapeutic potential.

Disease mutations in RyR1 and RyR2 have been linked to several disorders. Our crystal structures of the SPRY1 and Repeat12 domains allow direct mapping and interpretation of eight such mutations. Given the direct involvement of SPRY1 in FKBP binding, the question arises whether SPRY1 disease mutations directly affect FKBP binding. Indeed, we identified the N760D mutation (corresponding to the human N759D core myopathy mutation^43^), which is located close to the FKBP interface. The mutation results in a fourfold reduction in total FKBP binding at saturating levels, showing for the first time that a disease mutant can have a profound effect on the association with FKBP. Interestingly, the mutation seems to act by simply reducing the number of binding sites approximately fourfold, as if ∼75% of the SPRY1 domains are misfolded, but the remainder still available with nearly identical affinity. On the isolated SPRY1 domain, it decreases the yield of recombinant expression four- to eightfold, but the portion that can be purified is still well behaved and non-aggregating. This implies that the residue plays a role in folding of the SPRY1 domain, but once properly folded its role in maintaining this fold is less important.

## Methods

### Expression, purification and crystallization

Mouse RyR2 650-844 (SPRY1) and rabbit RyR1 857-1054 (Repeat12) were cloned into the pET28-HMT vector^4^. All four cysteines in Repeat12 were mutated to alanine by Quikchange (Stratagene) to facilitate crystallization. Proteins were expressed and purified using a strategy similar to the one for other RyR domains^4^, except that Repeat12 was purified by a Hiload SP Sepharose column (GE Healthcare) before a preparative Superdex 200. Selenomethionine (SeMet) labelled SPRY1 was produced using the modified autoinduction media^48^.

All crystals were grown at 4 °C using the hanging-drop method. SPRY1 was concentrated to 10 mg ml^−1^ and crystallized in 0.1 M sodium acetate (pH 5), and 0.6–0.9 M sodium sulphate. Repeat12 was concentrated to 10 mg ml^−1^ and crystallized in 0.1 M HEPES (pH 7), and 19% (w/v) PEG20,000. All crystals were harvested and flash cooled in the corresponding well solutions supplemented with 25–35% isopropyl alcohol.

### Data collection and structure determination

Diffraction data sets were collected at the Canadian Light Source beamline 08ID-1, the Stanford Synchrotron Radiation Light source beamline BL7-1, and Advanced Photon Source beamline 23-ID-D, and were processed with XDS^49^ and the HKL3000 package (HKL Research). Initial phases were calculated by MAD for SPRY1 and by iodide-SAD for Repeat12. Repeat12 crystals were soaked in 0.2–1 M NaI for 2 min to 1 h before freezing. The structures were refined with PHENIX^50^ using high-resolution native data sets at 1.21 and 1.55 Å, for SPRY1 and Repeat12, respectively. Both models were completed with iterative cycles of manual model building in COOT^51^ and refinement with PHENIX. B-factors have been refined anisotropically and hydrogens have been added in the riding positions for both structures. Data collection and refinement statistics are available in Table 1. The SPRY1 crystal structure contains two molecules in the asymmetric unit. All analysis has been performed with chain A. Coordinates and structure factors are available in the Protein Data Bank with accession codes 5C30 and 5C33. All structure figures were generated using UCSF Chimera^52^.

### Cryo-EM Docking

ADP_EM^35^ was used to dock the crystal structures of SPRY1, SPRY2 and Repeat12 into three different cryo-EM maps with EMDB entries 6,107, 2,751 and 2,807. The crystal structures were blurred to the reported resolutions for each entry (6.1, 4.8 and 3.8 Å, respectively), and docked with a default bandwidth setting of 16, no density cut-off, and with Laplacian filtering for SPRY1 and SPRY2. No Laplacian filtering was used for Repeat12, since this only yielded nonsense results. Laplacian filtering adds extra penalties for mismatches in surface features, and therefore does not work for the Repeat12 domains due to the poor density and lower resolution for the corner region of the RyR1 maps.

### MDFF

The RyR1 SPRY1 model was created by first substituting RyR1-specific residues into the RyR2 SPRY1 structure using MODELLER9.14 (ref. 53). Missing loops were built directly into the cryo-EM density maps using manual building and real-space refinement in COOT^51^. The resulting model was further optimized by constrained molecular dynamic simulations with NAMD2.10 (ref. 54) through the MDFF plugin^55^. The MDFF simulation was carried out in vacuum with restraints for secondary structure, chirality, and *cis*-peptide derived from the initial models. Each simulation was run for 20 ns with different grid force scaling parameters varying from 0.3 to 1. The convergence and accuracy of the simulations were assessed by the backbone r.m.s.d. with respect to the initial model and the correlation between model and map respectively. The Timeline plugin in VMD^56^ was used to create the per secondary structure cross-correlation coefficient plots.

### Fluorescence-based thermal shift assays

Protein stability was measured by fluorescence-based thermal shift assays^57^. 10 μl of protein (1 mg ml^−1^) was diluted to 50 μl with buffer (10 mM HEPES pH7.4, 150 mM KCl) containing SYPRO orange dye (Invitrogen). Thermal melts were performed using a DNA Engine Opticon 2 real-time PCR machine (Biorad), with the SYBR green filter. The temperature was increased from 25 to 95 °C with 0.5 °C steps and held constant for 15 s before data acquisition. Curves were normalized and the first derivatives were generated to calculate the melting temperatures.

### Full-length RyR1 cDNA cloning and expression

A RyR1 cDNA subclone flanked by *Nhe*I/*Age*I restriction sites encoding amino acids 1-1645 of rabbit RyR1 (ref. 58) was used for primer extension-driven mutagenesis to insert His_10_-tags into SPRY1. Forward and reverse oligonucleotide primers (IDT, Coralville, IA) each containing the His_10_-tag coding sequence, 5′-CAC–CAT–CAC–CAT–CAC–CAT–CAC–CAT–CAC–CAT-3′ as well as RyR-specific sequences annealing at cDNA positions corresponding to protein sequence 655 or 675 were paired with primers annealing at positions 515 and 802. Forward and reverse PCR products were amplified using Phusion high fidelity DNA polymerase (New England Biolabs, Ipswich, MA), combined with *Hpa*I/*Not*I-digested Nhe/Age subclone cDNA, and assembled using Gibson Assembly (New England Biolabs). The 675 loop scramble, F674A/L675A and N760D clones were created using unique 407 nucleotide GeneBlocks (IDT) each inserted between unique *Bgl*II/*Bsu36*I restriction sites in the Nhe/Age RyR1 subclone, using Gibson Assembly. After confirmation of proper sequence and reading frame using bidirectional DNA sequencing, His-tags, the 675 loop scramble and the site-directed mutations were cloned into the full- length RyR1 cDNA (for FRET analysis) or a GFP-RyR1 cDNA (for FKBP binding analysis) within the pCiNeo mammalian expression vector (Promega, Madison, WI). cDNAs expressed in HEK-293T cells using polyethylenimine-mediated transfection^59^ were tested in functional and FRET-based assays two days after transfection.

### Calcium imaging

Transfected HEK-293T cells expressing His-tagged RyRs were functionally tested for caffeine-induced Ca^2+^ release using Fluo-4 mediated intracellular Ca^2+^ imaging which we have previously described in full detail^59^.

### FRET imaging

Single-cysteine FKBP12.6 mutants were labeled with AF488 (donor), and Cy3NTA (acceptors) were synthesized^30^,^59^,^60^. Each His_10_-tagged RyR construct expressed in HEK-293T cells was analysed using FRET^33^,^58^ as summarized here. Cells were incubated overnight in FRET buffer (125 mM NaCl, 5 mM KCl, 6 mM glucose, 25 mM HEPES pH 7.6) containing 0.1% saponin, 10 nM AF488-FKBP and 3 μM Cy3NTA. Using epifluorescence microscopy, AF488-FKBP donor fluorescence was then quantified using a YFP cubeset from a Z-stack 60 microns in thickness taken before and after photobleaching the Cy3NTA acceptor using a ReAsH cubeset. FRET was then calculated using:




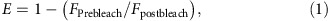




where, *E* represents the FRET efficiency and *F*_prebleach_ and *F*_postbleach_ indicate donor fluorescence intensities before and after acceptor photobleaching, respectively, as quantified from the Z-stack images using ImageJ version 1.45 m (NIH). These FRET efficiencies were then converted to intramolecular distances using:




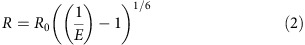




where *R* represents the donor/acceptor distance, *R*_0_ represents the Förster distance for the AF488/Cy3NTA pair (59 Å)^33^, and *E* represents the measured FRET efficiency. These distances were used for trilaterations and are given in Supplementary Fig. 13.

### Trilateration

We utilized AF488-FKBP donor positions that were determined computationally using a simulated annealing protocol^61^. These optimized AF488 donor locations and distances calculated from our measured FRET values were used to determine a locus in space corresponding to the position of Cy3NTA bound to the His^655^ tag using the trilateration method previously described^61^. This locus was visualized as a volume in Chimera relative to our SPRY1 atomic structure docked to the cryo-EM map with EMDB accession code 2807.

### FKBP binding assay

Relative FKBP binding to RyRs containing SPRY1 His_10_-tags or scrambles was determined by comparing the AF568-FKBP fluorescence intensity to the expression level of each construct, as determined from the fluorescence intensity of an N-terminally fused GFP, as shown before^58^. The ratio of AF568-FKBP fluorescence to GFP-mutant-RyR1 fluorescence measured under identical camera/gain settings was normalized to the corresponding ratio derived from cells expressing GFP–WT-RyR1. FKBP off-rate (*k*_off_) was measured after washing the AF568-FKBP loaded cells in imaging buffer supplemented with 1 μM unlabelled FKBP. Dissociation curves fitted using single-exponential decay kinetic analysis (Prism, v5.0 f) were used to derive *k*_off_ (1/*τ*_off_) values.

## Additional information

**Accession codes:** Atomic coordinates and structure factors files have been deposited in the Protein Data Bank under accession codes 5C30 and 5C33.

**How to cite this article:** Yuchi, Z. *et al*. Crystal structures of Ryanodine Receptor SPRY1 and tandem-repeat domains reveal a critical FKBP12 binding determinant. *Nat. Commun.* 6:7947 doi: 10.1038/ncomms8947 (2015).

## Supplementary Material

Supplementary Information Supplementary Figures 1-15 and Supplementary Table 1

## Figures and Tables

**Table 1:** Data collection and refinement statistics.

	**RyR2 SPRY1 (native)**		**RyR2 SPRY1 (MAD)**		**RyR1 Repeat12 (native)**	**RyR1 Repeat12 (SAD)**
*Data collection*						
Space group	P 2_1_ 2_1_ 2_1_		P 2_1_ 2_1_ 2_1_		P 3_1_ 2 1	P 3_1_ 2 1
Cell dimensions						
a, b, c (Å)	53.25 64.09 109.77		53.08 64.37 109.57		94.44 94.44 67.22	53.12 53.12 141.05
α,β,γ (°)	90 90 90		90 90 90		90 90 120	90 90 120
		**Peak**	**Inflection**	**Remote**		
Wavelength	1.033	0.975910	0.979518	1.377552	1.033	1.8965
Resolution (Å)	38.37–1.21 (1.25–1.21)	38.37–1.44	38.36–1.44	38.39–2.21	40.89–1.55 (1.61–1.55)	50–2.35 (2.41–2.35)
Rmerge	0.058 (0.915)	0.074	0.073	0.049	0.123 (1.509)	0.226 (1.252)
*I*/*σI*	17.81 (2.16)	20.22	20.56	39.33	13.82 (2.17)	17.46 (2.17)
Completeness (%)	99.9 (98.8)	99.5	99.3	99.6	99.2 (98.1)	99.9 (99.2)
Redundancy	7.0 (6.7)	8.8	13.4	13.1	11.9 (12.1)	20.3 (11.3)
						
Refinement						
Resolution (Å)	38.37–1.21				40.89–1.55	
No. reflections	114,712				50032	
*R*_work_/*R*_free_	14.33/17.25				14.91/16.45	
No. of atoms						
Protein	2951				1618	
Ligand/ion	9				8	
Water	479				388	
B-factors						
Protein	16.70				25.50	
Ligand/ion	29.60				21.60	
Water	29.40				41.60	
R.m.s.d.						
Bond lengths (Å)	0.015				0.011	
Bond angles (°)	1.59				1.35	

R.m.s.d., root mean-squared deviation.

One crystal was used for each structure. Highest resolution shell is shown in parenthesis.
