# Supplementary material for: Crystal structures of ryanodine receptor SPRY1 and tandem-repeat domains reveal a critical FKBP12 binding determinant
Source: Nat Commun. 2015 Aug 6;6:7947. doi: 10.1038/ncomms8947 (PMC4530471; doi:10.1038/ncomms8947)
Supplement: Supplementary Information — Supplementary Figures 1-15 and Supplementary Table 1 [file ncomms8947-s1.pdf]

Supplementary Information

Supplementary Figure 1

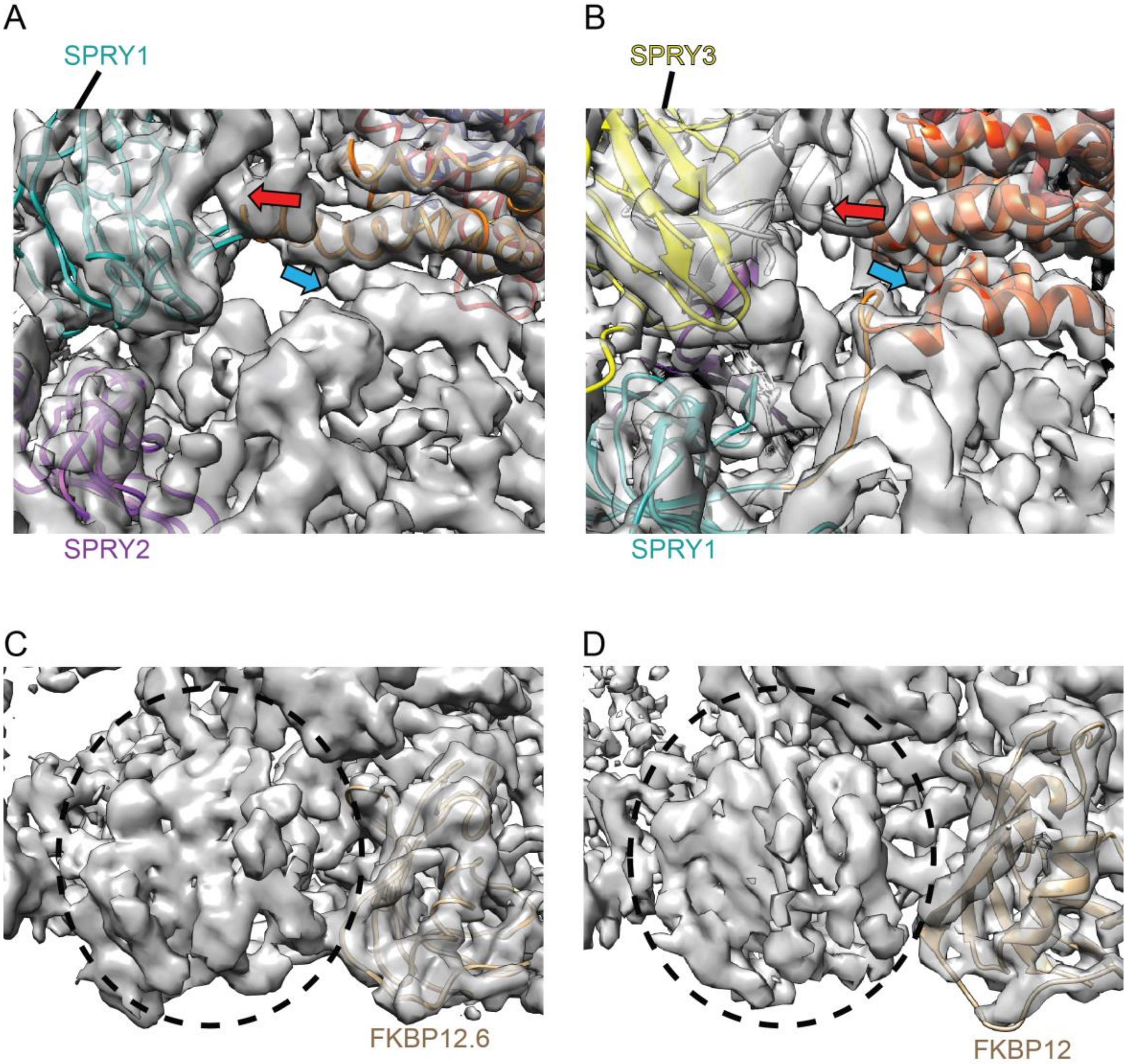

Ambiguity in the RyR1 cryo-EM maps. Details of the 4.8Å (panel **A**) and 3.8Å (panel **B**) maps of RyR1 showing two alternative pathways of connectivity after residues 590-600. Shown are cartoon representations for the corresponding deposited models, showing the previously crystallized domain C (red), and the following helices in the armadillo repeats (orange). The two possible pathways, which are visible in both maps, are indicated by red and blue arrows. Following the pathway to the left (red arrow) leads to the SPRY1 domain in the density on the left (panel A, cyan). Following the pathway downwards leads to extra helices in the region 600-630, and the SPRY1 domain near the bottom of panel B (cyan). In the latter case, the SPRY3 domain ends up in the density on the left (yellow). **C, D** Cryo-EM density of a domain next to FKBP12.6 or FKBP12 in the 4.8Å and 3.8Å maps, respectively. The dotted circle indicates an individual domain likely corresponding to a SPRY domain. In both cases, *de novo* tracing of the structure is clearly not possible. The fitted crystal structures for FKBP12.6 and FKBP12 are shown in cartoon representation (beige).

Supplementary Figure 2

A

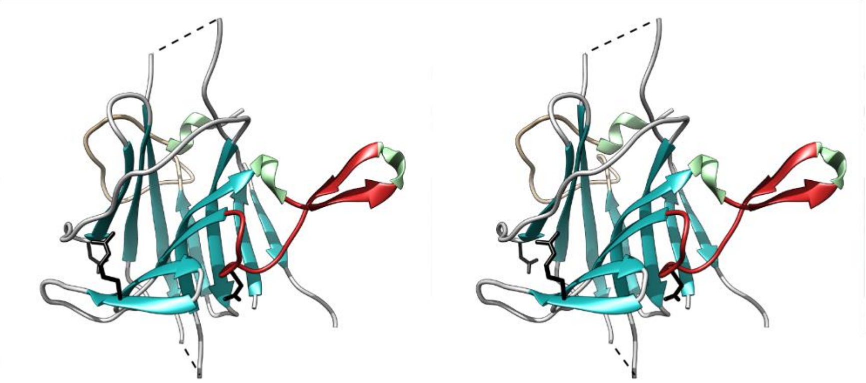

B

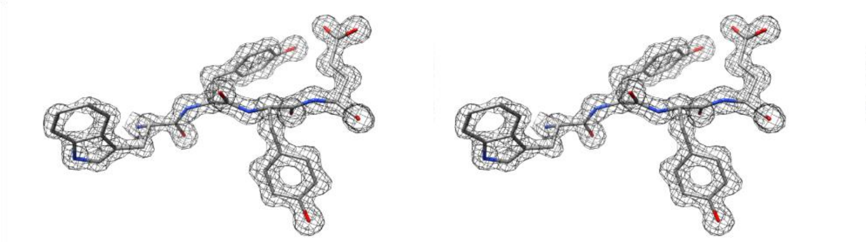

C

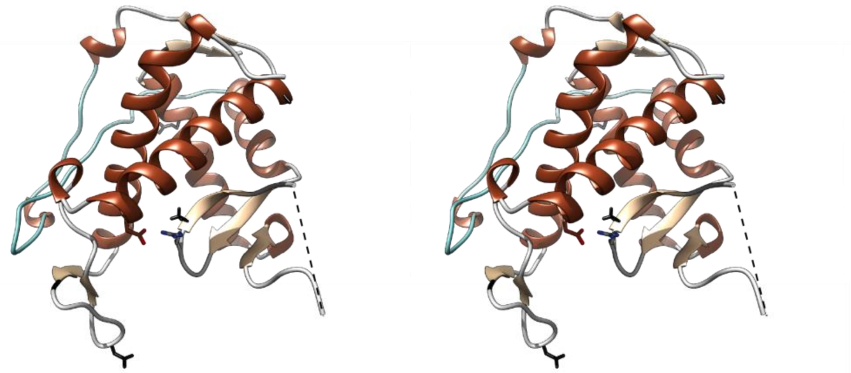

D

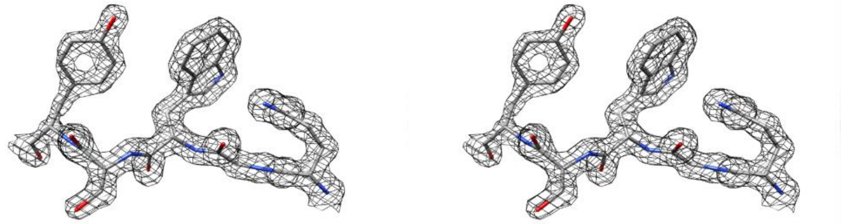

Cross-eyed stereo views of the RyR2 SPRY1 domain backbone trace (**A**) and 2mFo-DFc electron density (**B**), the RyR1 Repeat12 domain backbone trace (**C**) and 2mFo-DFc electron density (**D**). Densities are shown at 1.5σ cut-off values. Coloring scheme in panels A and C is the same as for Figures 2 and 3 in the main manuscript.

Supplementary Figure 3

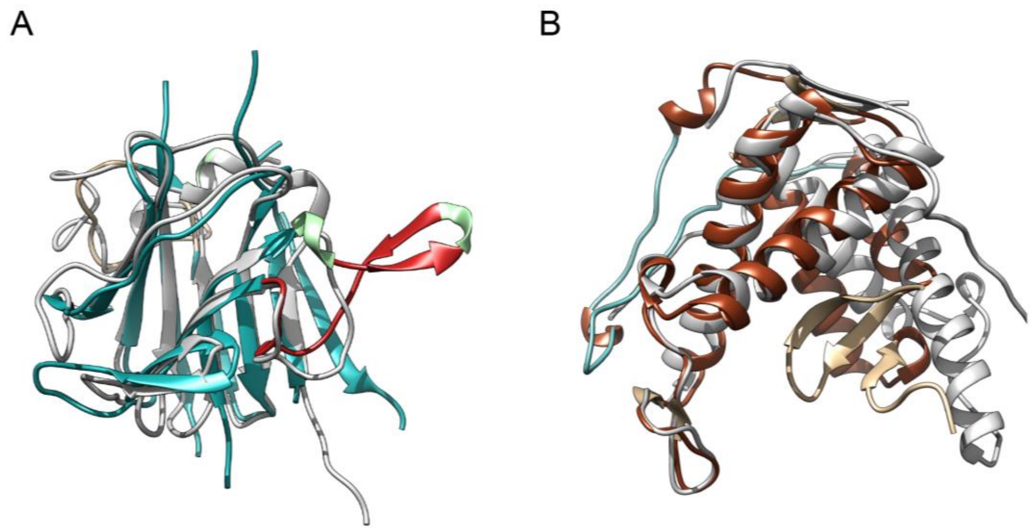

A. Superposition of the RyR2 SPRY1 domain (colors) with the RyR1 SPRY2 domain (grey). Although the core strands are preserved, the loops vary in length and conformation, and the SPRY2 domain lacks the finger. **B.** Superposition of the RyR1 Repeat12 domain (colors) and the RyR1 Repeat 34 (i.e. phosphorylation) domain (grey). The superposition is done using the first repeats only, indicating the different angles between the repeats. The U-lid and three-stranded β-sheet are not present in the phosphorylation domain, indicating that the latter is not a good template to produce homology models of the Repeat12 domain.

Supplementary Figure 4

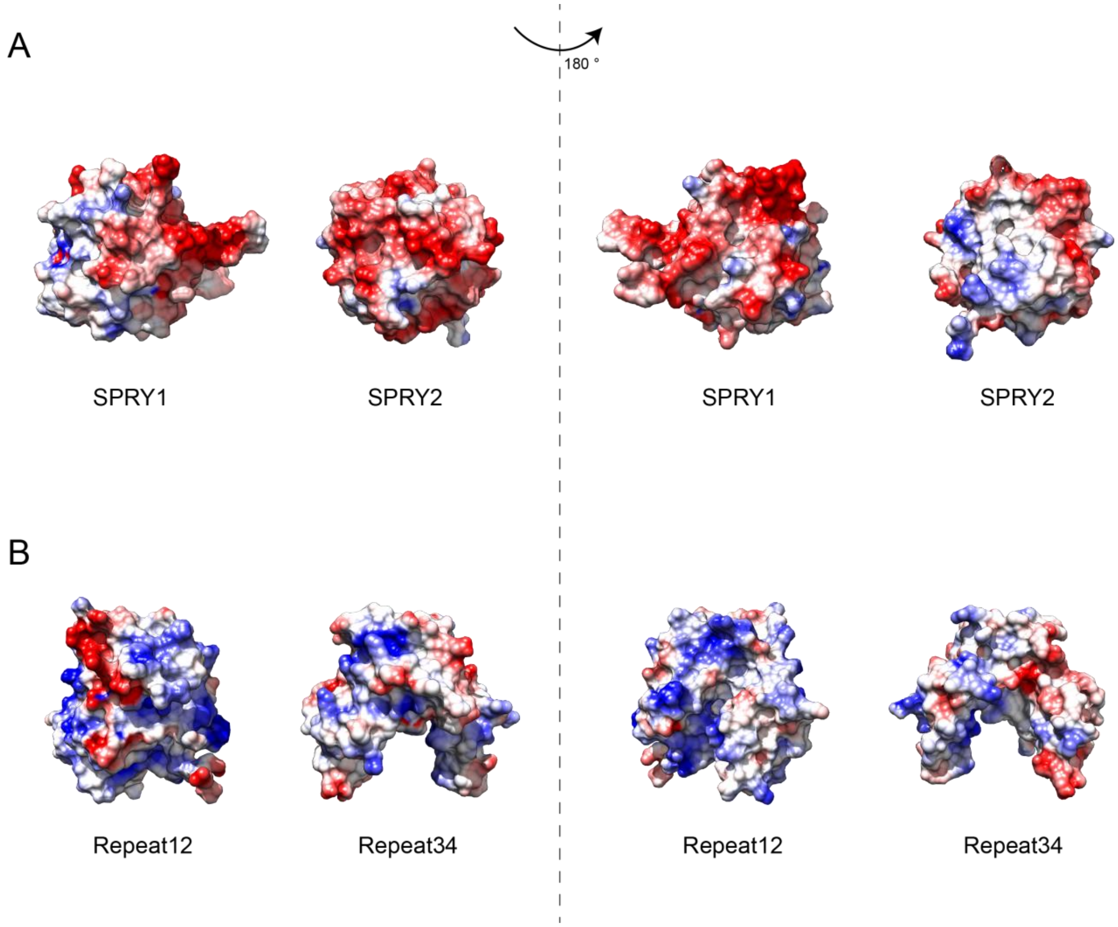

Comparison of the Van der Waals surfaces of SPRY1 versus SPRY2 (**A**) and Repeat12 versus Repeat34 (**B**). The electrostatic potentials are shown, with negative potentials in red, and positive potentials in blue. Flexible loops, for which no electron density was observed, are not included. Overall, there are distinct shape differences between SPRY1 and SPRY2, which are the result of several loops with different lengths and conformations. Repeat34 forms a horseshoe-shaped structure, which is not observed for Repeat12 due to the presence of a three-stranded β-sheet that fills up the space. The repeat domains are predominantly positively charged.

Supplementary Figure 5

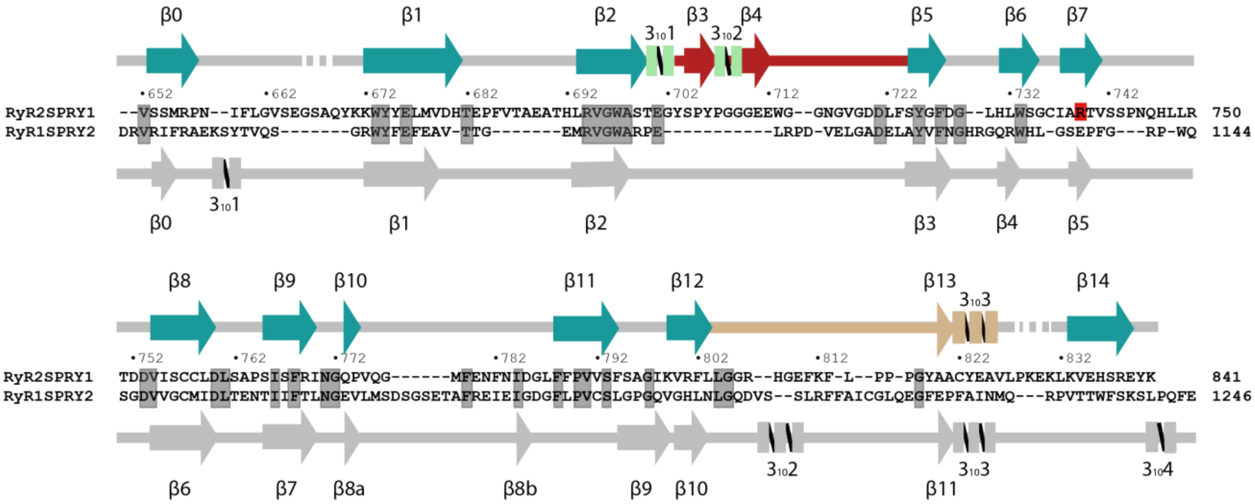

Structure-based sequence alignment of the SPRY1 and SPRY2 domains. Secondary structure elements are indicated above and below. Stretches that are part of the crystallized constructs but that displayed no density are shown as dotted lines. The ‘finger’, formed by two antiparallel β-strands (red) pointing away from the SPRY core is unique for SPRY1.

Supplementary Figure 6

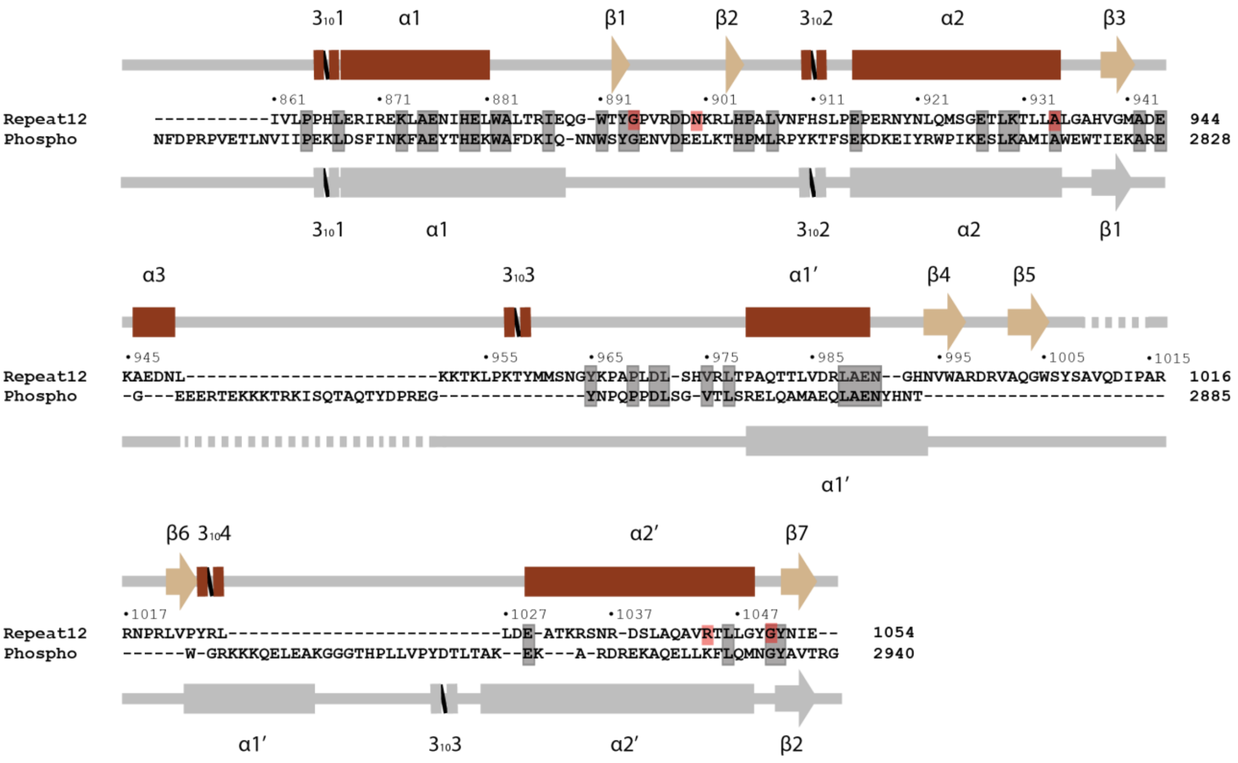

Structure-based sequence alignment of the RyR1 Repeat12 and Repeat34 (phosphorylation) domains. Secondary structure elements are shown above and below. The first repeat of each domain corresponds quite well, but there is almost no structural homology within the inter-repeat linker, and the second repeat in Repeat12 has a shortened α1′ helix, followed by a unique three-stranded β-sheet (strands $\beta_{4\text{-}6}$).

Supplementary Figure 7

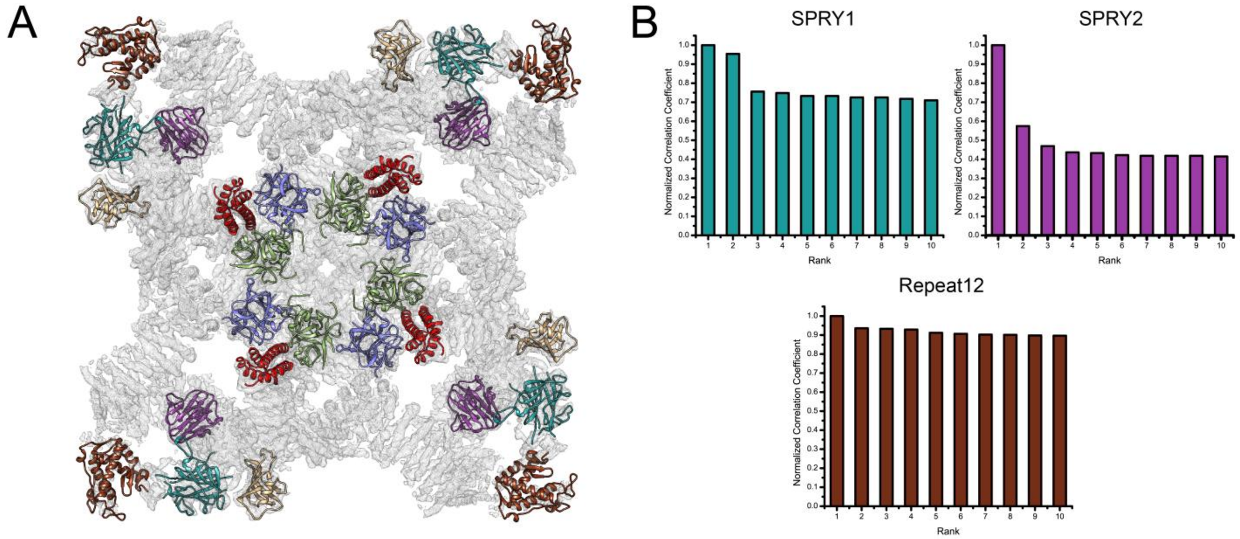

A, Positions of SPRY1 (cyan), Repeat12 (brown) and SPRY2 (magenta) in the 4.8Å cryo-EM map of CIP-treated rabbit RyR1 with bound FKBP12.6 (EMDB 6107). Also shown are the positions for the N-terminal domains A (blue), B (green) and C (red), as well as FKBP12.6 (tan). **B,** Normalized correlation coefficients for the top ten hits for docking of the crystal structures of SPRY1, SPRY2, and Repeat12.

Supplementary Figure 8

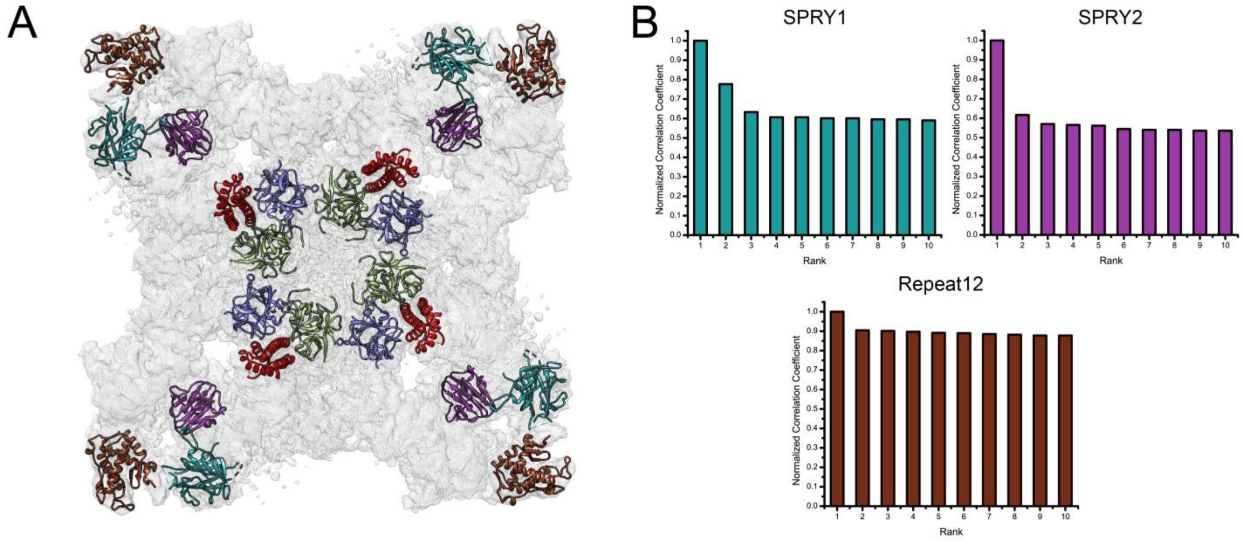

A Positions of the SPRY1 (cyan), Repeat12 (brown) and SPRY2 (magenta) in the 6.1Å cryo-EM map of rabbit RyR1 (EMDB 2751). Also shown are the positions for the N-terminal domains A(blue), B (green) and C (red). No FKBP was observed in this map. **B** Normalized correlation coefficients for the top ten hits for docking of the crystal structures of SPRY1, SPRY2, and Repeat12.

Supplementary Figure 9

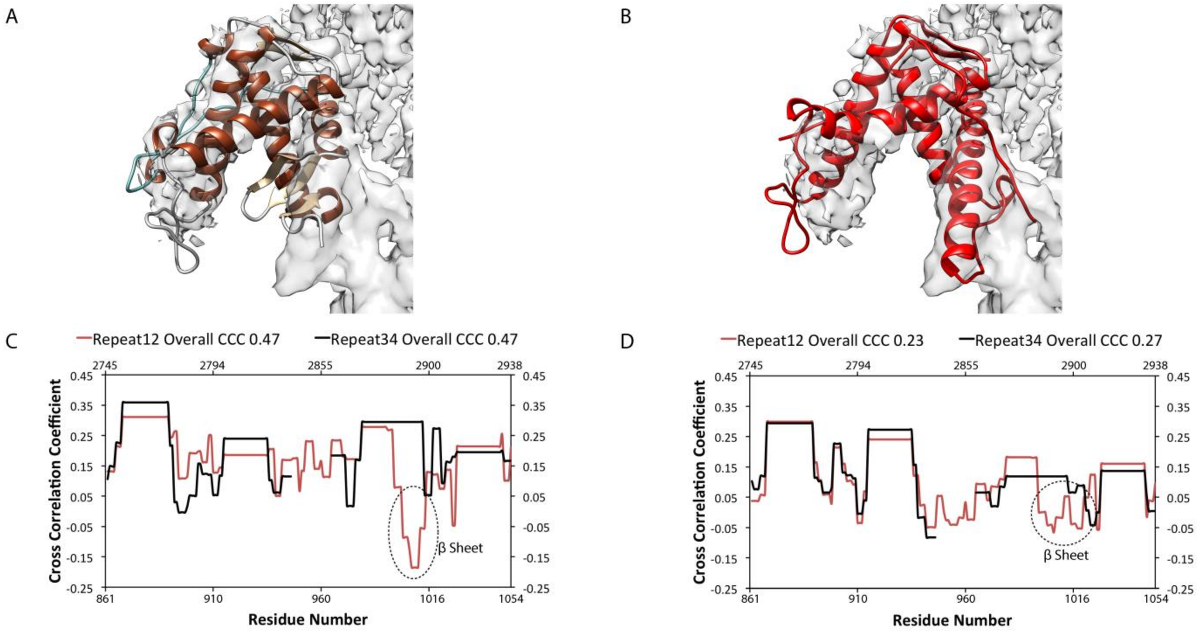

A, Local fit of the Repeat12 domains in the corner of the 3.8Å map. There is a visible mismatch between the crystal structure and the electron density, which does not show the three-stranded β-sheet. **B,** Docked position of the top hit for the Repeat34 (phosphorylation) domain in the 3.8Å map. **C,D** Cross correlation coefficients (CCC) for Repeat12 and Repeat34 in the corner (**C**) and turret (**D**) positions of the 3.8Å map, per secondary structure element as calculated by VMD. The location of the β-sheet, which has the lowest CCC in the corner position, is indicated.

Supplementary Figure 10

A **B**

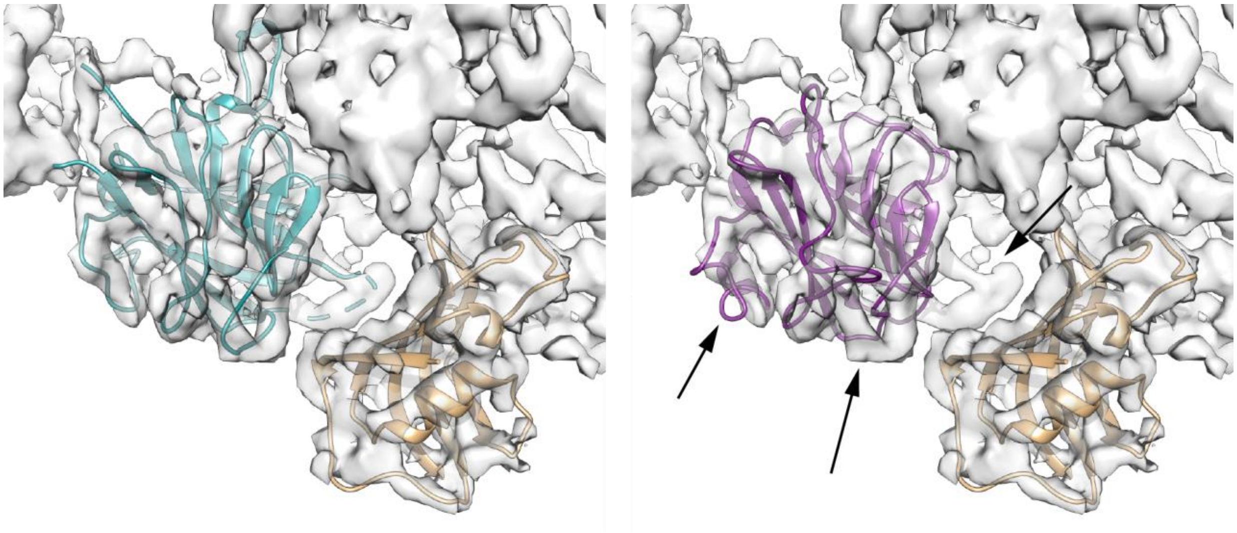

C **D**

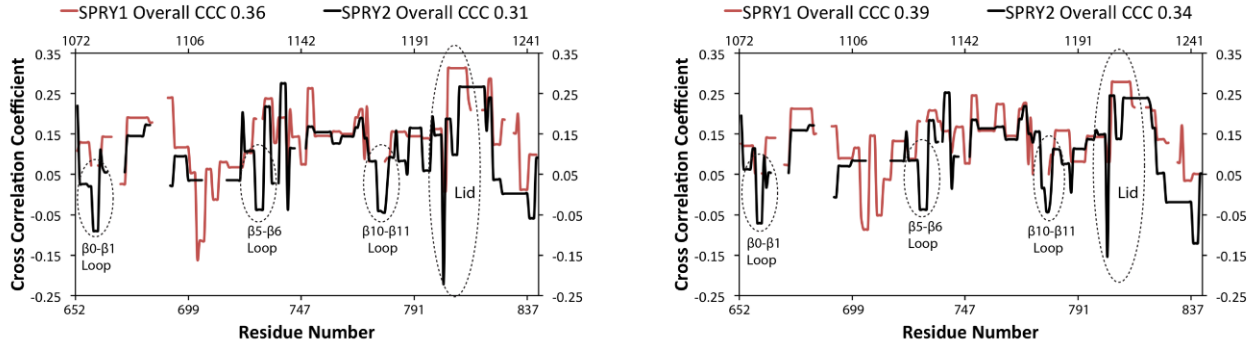

A,B, Side-by-side comparison of fits for crystal structures of SPRY1 (cyan, left) and SPRY2 (magenta, right) into the globular density next to FKBP12.6 (beige). The electron density is for the 4.8Å map (EMDB 6107), which shows the clearest density for a loop pointing to FKBP12.6 (dotted cyan line). The SPRY1 domain visually fits much better in the density than SPRY2. Arrows on the right panel show several loops that do not fit well for SPRY2 (arrows). Most importantly, a flexible loop in SPRY1 has clear density in the map next to FKBP12 (dotted cyan lines in the left panel), suggesting it becomes ordered upon FKBP12/12.6 binding. The corresponding loop in SPRY2 has a minimal length to link the β-strands and cannot extend to the FKBP12 or FKBP12.6 surface. **C,D,** Correlation coefficients per secondary structure element for fits of SPRY1 and SPRY2 into the position next to FKBP, for the 3.8Å (**C**) and 4.8Å (**D**) maps. SPRY2 loops that do not fit well are highlighted. Overall CCC values determined by VMD are also shown.

Supplementary Figure 11

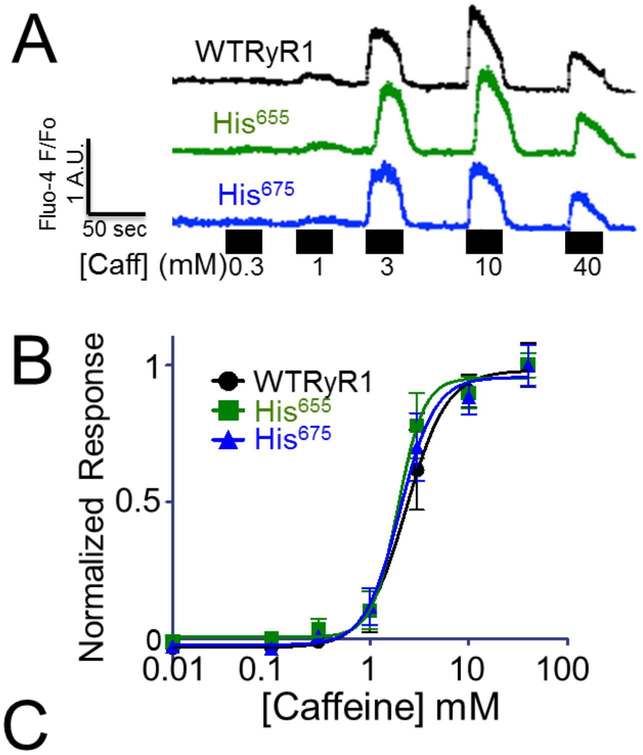

C

Construct	*n*	EC_{50} (mM)	95% C.I. (mM)
WTRyR1	26	2.3	1.9-2.7
His655	28	1.9	1.7-2.2
His675	29	2.0	1.8-2.3

Functional characterization of His_{10}-tagged full-length RyR1 constructs using Fluo-4 based intracellular Ca^{2+} imaging. This figure is related to Figure 5 in the main manuscript. **A,** Representative caffeine-induced intracellular Ca^{2+} transients for indicated constructs expressed in HEK-293T cells are shown. A graded series of caffeine concentrations was perfused at the time intervals indicated by the black bars. **B,** The concentration dependence of caffeine activation of the indicated RyR1 constructs is shown. Values represent mean $\pm$ SEM. **C,** Summary of mean EC_{50} and 95% confidence interval (C.I.) of the mean for the number of cells analyzed (*n*). No significant differences in mean EC_{50} were observed as determined by one-way ANOVA followed by a Dunnett's post-test ($p<0.05$).

Supplementary Figure 12

A

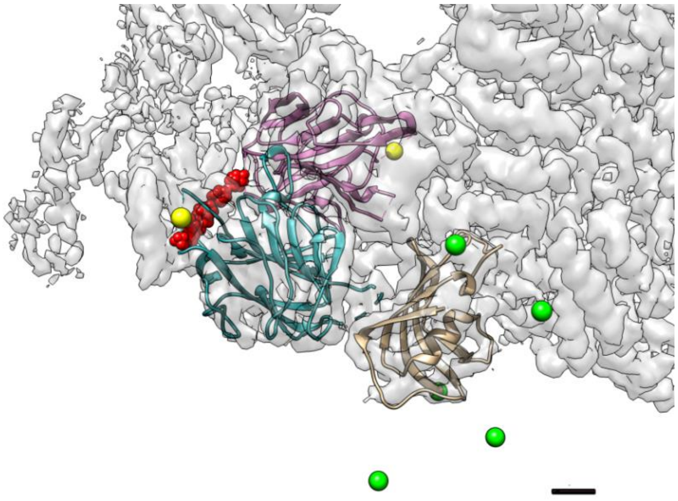

B

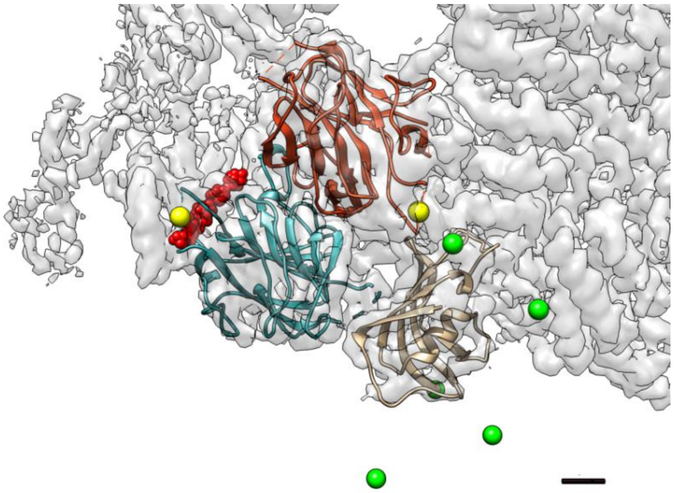

The FRET-based trilaterated locus of acceptor attached to the His^{655} tag is incompatible with alternative locations of the SPRY1 domain. This figure is related to Figure 5 in the main manuscript. Shown are FKBP12 (beige), SPRY1 in the top docked position (cyan), or in the position where SPRY2 docks, which is hit #2 in the docking (panel A, purple), or in the position where SPRY3 is expected to be, corresponding to hit #3 in the SPRY1 docking (panel B, orange). The small red spheres correspond to the trilateration locus. Green spheres correspond to the effective locations of the five FRET donors covalently bound to FKBP. Yellow balls correspond to the position of the His655 loop used for the trilateration. Only the top docked location has the His655 loop fall directly within the trilateration area. The alternative locations in panels A and B result in the His655 loop being 43Å and 47Å away from the closest trilateration point, respectively.

Supplementary Figure 13

A

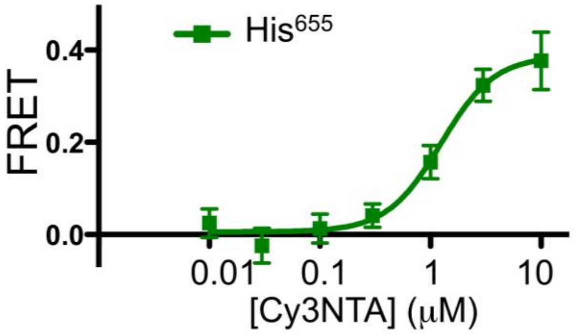

B

His^{655}			
FKBP Labeled Site	*n*	FRET	R (Å)
D14	36	0.33 ± 0.05	66 ± 4.1
D32	27	0.52 ± 0.07	58 ± 3.6
D44	41	0.44 ± 0.11	61 ± 3.8
D49	31	0.25 ± 0.03	71 ± 4.4
D85	38	0.31 ± 0.05	67 ± 5.0

FRET-based measurement of intramolecular distances between FKBP and SPRY1 in full-length RyRs. This figure is related to Figure 5 in the main manuscript. **A,** The Cy3NTA concentration dependence of FRET from AF488-D44-FKBP12.6 to Cy3NTA bound to His^{655} is shown. Fractional occupancy of 3 μM Cy3NTA binding (85%) was used to correct static FRET measurements (Fig. 5) used for trilateration. **B,** Summary of FRET measurements from donors bound to FKBP at the indicated sequence positions to acceptor attached to His^{655}. FRET values were converted to donor/acceptor distances (Equation 2, R_0 = 59 Å), which were used to trilaterate Cy3NTA bound to His^{655} Spheres of radius R (centered at the indicated donor positions), and skin thickness 12.5% of R were intersected in order to trilaterate the position of Cy3NTA bound to His^{655}. FRET and R values are indicated as means ± SEM. *n* indicates the number of measurements.

Supplementary Figure 14

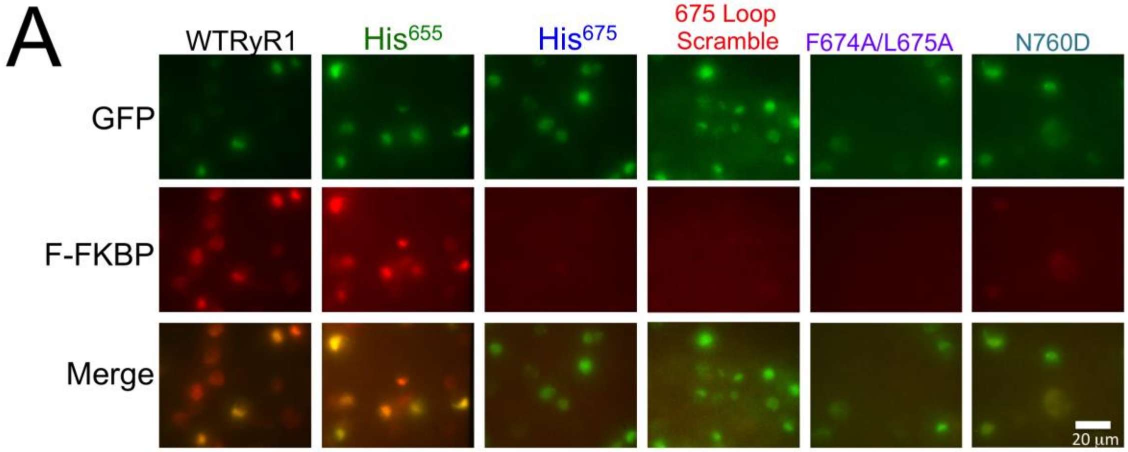

B

RyR1 Construct	B_{max} (Fraction of WTRyR1)		K_d (nM)		Dissociation Half Life (min^{-1})	
	Mean ± SEM	*n*	Mean	*n*	Mean	*n*
WTRyR1	0.98 ± 0.09	29	1.63	17-29	131.1	13
His^{655}	0.73 ± 0.09	41	0.87	26-41	184	15
His^{675}	0.05 ± 0.01	37	>10	23-37	7.4	9
675 Loop Scramble	0.03 ± 0.01	23	>10	17-25	35.0	6
F674A/L675A	0.04 ± 0.01	16	>10	16-27	11.5	5
N760D	0.26 ± 0.05	22	1.02	20-30	70.0	11

FKBP binding to full-length GFP-RyR1 fusion constructs with mutations in the SPRY1 domain. **A**, HEK293T cells expressing GFP-tagged RyRs containing His_{10} or scrambled sequences in the SPRY1 domain are shown after equilibration in saturating (10 nM) D49-AF568-FKBP (F-FKBP). GFP (top panels), F-FKBP fluorescence (middle) and a merged image of the two channels (bottom) are shown. **B**, Summary data corresponding to F-FKBP binding analyses from Fig. 7 are shown. F-FKBP:RyR1 B_{max} for each construct is normalized to the B_{max} of WT GFP-RyR1. K_d values were not determined for the His^{675}, 675 loop scramble, and F674A/L675A constructs, where no saturable binding was observed (Fig. 7).

Supplementary Figure 15

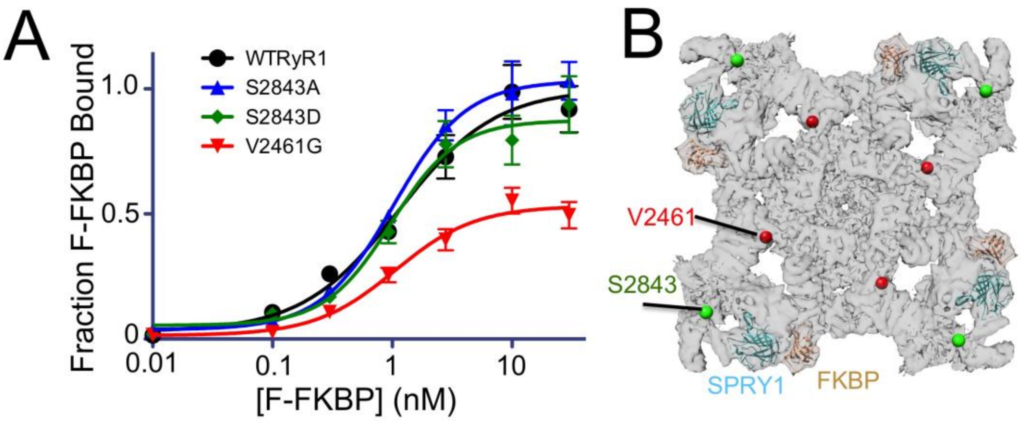

C

RyR1 Construct	B_{max} (Fraction of WTRyR1)		K_d (nM)	
	Mean + SEM	*n*	Mean	*n*
WTRyR1	0.98 + .11	29	1.13	23-30
S2843A	0.98 + 0.13	19	1.02	15-22
S2843D	0.80 + 0.09	26	0.98	12-26
V2461G	0.55+ 0.05	60	1.05	18-64

Analysis of FKBP binding to GFP-RyR1 fusion constructs containing mutated putative FKBP binding sites. **A**, Concentration dependence of AF568-FKBP binding to the indicated GFP-RyR1 mutants expressed in HEK293T cells relative to WT GFP-RyR1 (black symbols). **B**, Location of V2461 (red sphere) and S2843 (green sphere) in the 3.8 Å RyR1 cryo-EM structure (gray). **C** Summary data from FKBP binding analyses for the indicated GFP-RyR1 constructs are shown. F-FKBP:RyR1 B_{max} for each construct is normalized to the B_{max} of WT GFP-RyR1. *n* indicates the number of cells analyzed.

Supplementary Table 1

3J8H (3.8 Å)		MDFF EMD-2807 (3.8 Å)		MDFF EMD-6107 (4.8 Å)	
**SPRY1**	**FKBP12**	**SPRY1**	**FKBP12**	**SPRY1**	**FKBP12.6**
		**Pro673**	Arg71		
**Phe674**	Arg40<br>Arg71<br>Glu102	**Phe674**	Arg40<br>Arg71	**Phe674**	His25<br>Arg40<br>Arg71
				**Leu675**	Arg40
**Ala677**	Arg40<br>Asp41	**Ala677**	Arg40	**Ala677**	Arg40
**Ala679**	Arg71			**Ala679**	Arg40
		**Leu719**	Ile7<br>Ser8		
		**His736**	Pro9	**His736**	Ser8
**Leu737**	Ile7<br>Ser8	**Leu737**	Ser8<br>Arg71	**Leu737**	Ile7<br>Ser8

Van der Waals interactions at the SPRY1-FKBP interface. Table comparing residues predicted to be in Van der Waals contact as analyzed by UCSF Chimera (VDW surfaces within 0.4Å from one another). Shown are the SPRY1 residues and the contacting FKBP residues in the published 3.8Å cryo-EM model (left), and the results of MDFF experiments in the 3.8Å (middle) and 4.8Å maps (right).
